# Interspecific Variation in the Unsaturation Level of Seed Oils Were Associated With the Expression Pattern Shifts of Duplicated Desaturase Genes and the Potential Role of Other Regulatory Genes

**DOI:** 10.3389/fpls.2020.616338

**Published:** 2021-01-13

**Authors:** Mengli Wang, Lexuan Gao, Gengyun Li, Chengchuan Zhou, Jinjing Jian, Zhen Xing, Yuguo Wang, Wenju Zhang, Zhiping Song, Yonghong Hu, Ji Yang

**Affiliations:** ^1^Ministry of Education Key Laboratory for Biodiversity Science and Ecological Engineering, Institute of Eco-Chongming (IEC), Fudan University, Shanghai, China; ^2^Institute of Plant Physiology and Ecology, Chinese Academy of Sciences, Shanghai, China; ^3^Tibet Agricultural and Animal Husbandry University, Linzhi, China; ^4^Shanghai Key Laboratory of Plant Functional Genomics and Resources, Shanghai Chenshan Botanical Garden, Shanghai, China

**Keywords:** seed oil, degree of unsaturation, regulatory gene, gene duplication, conserved co-expression

## Abstract

Seed oils are of great economic importance both for human consumption and industrial applications. The nutritional quality and industrial value of seed oils are mostly determined by their fatty acid profiles, especially the relative proportions of unsaturated fatty acids. Tree peony seed oils have recently been recognized as novel edible oils enriched in α-linolenic acid (ALA). However, congeneric species, such as *Paeonia ostii* and *P. ludlowii*, showed marked variation in the relative proportions of different unsaturated fatty acids. By comparing the dynamics of fatty acid accumulation and the time-course gene expression patterns between *P. ostii* and *P. ludlowii*, we identified genes that were differentially expressed between two species in developing seeds, and showed congruent patterns of variation between expression levels and phenotypes. In addition to the well-known desaturase and acyltransferase genes associated with fatty acid desaturation, among them were some genes that were conservatively co-expressed with the desaturation pathway genes across phylogenetically distant ALA-rich species, including *Camelina sativa* and *Perilla frutescens*. Go enrichment analysis revealed that these genes were mainly involved in transcriptional regulation, protein post-translational modification and hormone biosynthesis and response, suggesting that the fatty acid synthesis and desaturation pathway might be subject to multiple levels of regulation.

## Introduction

Plant storage oils are an essential part of the human diet, providing a number of nutrients the body needs including essential fats and vitamins (Damude and Kinney, 2008). Plant oils are also utilized as a major source of calories in our food supply and as feedstocks for non-food uses such as soaps and polymers (Dyer et al., 2008). Due to the concern on the depletion of fossil fuels and the environmental problems caused by the use of fossil fuels, plant oils have recently attracted considerable attention as cleaner, renewable, and sustainable sources for biofuel production (Durrett et al., 2008).

Plant seed oils consist principally of triacylglycerols (TAG’s) and contain both saturated and unsaturated (mono- and polyunsaturated) fatty acids, with the content and fatty acid (FA) composition varying considerably across plants (Voelker and Kinney, 2001). Variations in the ratios of saturated to unsaturated fatty acids (SFA: UFA), and monounsaturated to polyunsaturated fatty acids (MUFA: PUFA) have been widely detected in different plants (Zhang et al., 2015; Ohlrogge et al., 2018). The level of fatty acid unsaturation of oil can directly affect its value and utilization. Polyunsaturated fatty acids, such as linoleic acid (LA), and α-linolenic acid (ALA), are considered to be critical nutrients for human health (Damude and Kinney, 2008), but they are undesirable while being used as biofuel and chemical feedstocks because of their instability in processing, storage and use (Kodali, 2002). The requirements on different optimal fatty acid compositions for different uses have directly led to the growing interest in identifying genetic factors responsible for the variation in seed oil composition to better understand how the relative proportions of fatty acids are regulated in the seed oils derived from different plants.

A considerable amount of research has been conducted to identify genes potentially associated with the differential accumulation of unsaturated fatty acids in seed oils for optimizing the proportions of fatty acids through biotechnology for different industrial and nutrition goals (Rao et al., 2008; Haslam et al., 2013; Chen et al., 2014). Previous studies, however, have mostly focused on the genes encoding for three desaturases, i.e., stearoyl-ACP desaturase (SAD), fatty acid desaturases 2 (FAD2), and fatty acid desaturases 3 (FAD3) (Thambugala et al., 2013; Rajwade et al., 2014, 2016). These three enzymes catalyze stepwise desaturation of fatty acids during oil biosynthesis and determine the relative proportions of three major unsaturated fatty acids, oleic acid (OA), LA and ALA, in seeds. Although a great deal of evidence has shown the conserved roles of SAD, FAD2, and FAD3 across plant species, as well as the positive relations between high levels of fatty acid unsaturation and the upregulation of *SAD*, *FAD2*, and *FAD3* genes in developing seeds (Ohlrogge and Browse, 1995), it remains elusive how these genes are differentially regulated in different plants so as to collectively control the ratios of SFA: UFA and MUFA: PUFA. Duplicate desaturase genes with altered expression patterns during seed development have also been detected in many plants (Cao et al., 2013; Wang and Liu, 2014; Yurchenko et al., 2014; Han et al., 2017). It is unclear, however, whether the differential transcriptional activities of duplicates are responsible for the differential accumulation of PUFAs in different plants and to what degree the duplicates have diverged within and between species. The question still remains: which genes other than desaturase genes are temporally expressed corresponding to the dynamic synthesis of unsaturated fatty acids in developing seeds, regulating the transcription and catalytic activity of desaturation enzymes and potentially contributing to the variation in unsaturated fatty acids accumulation in different plants. The newly developed methods of genome wide association studies (GWAS) and genome-wide transcription analysis allow for a more efficient discovery of additional genes involved in fatty acid synthesis and desaturation (Li et al., 2013; Branham et al., 2015; Menard et al., 2017; Zhao et al., 2019).

As a traditional ornamental and medicinal plant in China, tree peony has attracted wide attention in the recent past for its relatively high oil content (27%) in seeds and high ratio of unsaturated fatty acids (over 90%) (Li S. et al., 2015). The oil of tree peony has now been recognized as novel edible oil enriched in omega-3 polyunsaturated fatty acid (Han et al., 2016). Comparative studies of oil content and fatty acid composition have revealed significant variations in the relative proportions of three major unsaturated fatty acids among different *Paeonia* (section *Moutan* DC) species. The polyunsaturated fatty acid ALA seems to be the dominant component in the oils of the species from the subsection *Vaginatae*, while the monounsaturated fatty acid OA is predominant in the species of the subsection *Delavayanae* (Yu et al., 2016). Genes associated with lipid accumulation and polyunsaturated fatty acid synthesis have been cloned and characterized in some tree peony species (Sun et al., 2018; Xiu et al., 2018; Yin et al., 2018). Gene expression patterns associated with differential accumulation of unsaturated fatty acids have also been examined between *P. rockii* (subsection *Vaginatae*) and *P. lutea* (subsection *Delavayanae*) (Zhang Q. et al., 2018; Zhang et al., 2019). However, their results showed that the predominant unsaturated fatty acid was ALA instead of OA in the oil of *P. lutea*, while multiple studies have shown that OA was predominated over LA and ALA in *P. ludlowii*, another species of the subsection *Delavayanae* (Han et al., 2016; Yu et al., 2016; Xiu et al., 2018). Differences in the ALA, LA, and OA content proportions are in fact indicative of the degree of unsaturation of different seed oils. Comparing the dynamics of fatty acid accumulation and gene expression between congeneric species with similar oil content but varied fatty acid composition can provide valuable clues for identifying genes potentially involved in the regulation of fatty acid desaturation.

In this study, we characterized the fatty acid profiles and the time-course gene expression patterns of *P. ostii* and *P. ludlowii*. Based on a comprehensively comparative analysis of homologous genes in sequence divergence, differential expression patterns and their co-expression relationships associated with phenotypic variation, we identified genes encoding desaturases and acyltransferases that were differentially expressed between two species in developing seeds. Network-based comparative transcriptome analysis of the data from the species of *Paeonia* and two other ALA-rich plants, *Camelina sativa* and *Perilla frutescens*, revealed genes potentially involved in regulating ALA biosynthesis and accumulation in plants.

## Materials and Methods

### Plant Materials

*P. ostii* was grown in Shanghai Chenshan Botanical Garden, Shanghai (31°4′52″N, 121°10′14 ″E). *P. ludlowii* was collected from Linzhi, Tibet (29°20′21.9″N, 94°22′35.8″E). Seeds were harvested every 10 days after flowering (DAF), spanning a total of 120 days. Three samples were collected for each species at each developmental stage. Samples used for FA analysis were dried to a constant weight at 60°C, while those used for RNA extraction were soaked in RNAlater solution and stored at −40°C prior to RNA extraction. Young stems and mature leaves were also collected and used for comparative analysis.

### Lipid Extraction and Fatty Acid Composition Determination

Lipid extraction and measurement of FA composition were conducted as previously described (Yu et al., 2016). Dried seeds were ground to fine powder. 0.2 g of the dried powder in a 3 mL mixture of 1:2 chloroform: methanol (v/v) was used to extract lipid. After fatty acid methylation, fatty acid methyl esters were analyzed by GC-MS (GC7890/MS5975, Agilent) equipped with a HP-88 capillary column (60 m, 0.25 mm, 0.2 μm, Agilent), using nonadecanoic acid as the internal standard. Omega-6 and omega-3 desaturation efficiencies (DE’s) were estimated by calculating the proportion of available substrate that was converted to product(s) as described (Menard et al., 2017): omega-6 DE = (18:2 + 18:3)/(18:1 + 18:2 + 18:3) and omega-3 DE = 18:3/(18:2 + 18:3).

### Total RNA Extraction, cDNA Library Construction, and High Throughput Sequencing

Based on the dynamic pattern of oil accumulation, five stages of seed development (0, 40, 50, 60, and 100 DAF) were selected for transcriptome sampling. Total RNA’s were extracted with TIANGEN RNA Prep Plant kit (Tiangen Biotech Co., Ltd.), following the manufacturer’s protocol. The quality and quantity of obtained RNA’s were determined with Nanodrop 2000C spectrophotometry (Thermo scientific) and 2100 Bioanalyzer (Agilent Technologies). RNA samples from five developmental stages of three replicates were pooled for each species and used for *de novo* transcriptome sequencing and assembly, to obtain comprehensive data on expressed gene sequences. cDNA library construction and Illumina sequencing were conducted at Beijing Genomics Institute (BGI) under the Illumina manufacturer’s instructions (Illumina). In brief, mRNA was isolated from total RNA using Oligo (dT), and subsequently treated with fragmentation buffer. The short-fragmented mRNA was then utilized as template to generate first-strand cDNA. Followed by second strand cDNA synthesis, end reparation, adaptor connection and PCR amplification, the cDNA libraries were sequenced on an Illumina HiSeq 4000. To establish a temporal map of gene expression for each species, RNA’s from the five developmental stages of three replicates were also individually used for cDNA library construction and sequencing. A total of fifteen independent cDNA libraries were generated for each species. The sequence raw data from this study have been submitted to the NCBI Sequence Read Archive (SRA)^1^ under the BioProject ID PRJNA602603.

### *De novo* Assembly, Functional Annotation, and Expression Quantification

After quality filtering, *de novo* transcriptome assembly was performed using Trinity (Grabherr et al., 2011). The software TGICL (Pertea et al., 2003) was then used to cluster the assembled transcripts to generate a single non-redundant unigene set for each species.

The BLASTx program was used to align the assembled unigenes against the NCBI non-redundant protein (Nr) database^2^, the Swiss-Prot protein sequence database^3^ and the *Arabidopsis* protein database at the *Arabidopsis* Information Resource (TAIR^4^) with an E-value threshold of 10^–10^. Functional annotation by Gene Ontology (GO^5^) terms were obtained using the Blast2GO program (Conesa et al., 2005). In addition, we obtained metabolic pathway annotations for each hit by searching against the Kyoto Encyclopedia of Genes and Genomes (KEGG^6^) pathway database (Ogata et al., 1999) using the BLASTx program with an *E*-value cut off of 10^–10^.

The clean reads generated from 15 independent cDNA libraries were separately aligned to the *de novo* assembled transcriptome for each species, respectively. The program RSEM (Li and Dewey, 2011) was used for estimating the expression levels of each unigene, normalizing into Fragments Per Kilobase per Million reads mapped (FPKM). The NOISeq R package was used to screen genes differentially expressed at different stages of seed development (Tarazona et al., 2011). Hierarchical clustering of genes was performed using the hclust function in R v.3.5.3 (Ihaka and Gentleman, 1996). The expression stoichiometries of oil accumulation-related genes were calculated with the methods used in previous studies (Troncoso-Ponce et al., 2011). The coefficient of variation (CV) was calculated to evaluate the expression consistence among growth stages and species.

To validate the reliability of the gene expression data obtained by RNA-Seq, the expression levels of genes of interest were measured by real-time quantitative reverse transcription polymerase chain reaction (RT-qPCR) with the LightCycler 96 system (Roche) using SYBR Green PCR Master Mix (TAKARA). Three independent biological replicates and three technical replicates were performed for each reaction. Based on previous studies (Li S.S. et al., 2015; Liu et al., 2015) and the coefficient of variation of expression level, a total of 5 candidate reference genes (ubiquitin, PUF1639, RPS9, Unigene15510_F/Unigene12802_D, and CL5272.Contig1_F/Unigene12410_D) were evaluated for the normalization of RT-qPCR. Three widely-used programs, GeNorm3.5 (Vandesompele et al., 2002), BestKeeper (Pfaffl et al., 2004), and NormFinder (Andersen et al., 2004), were used to rank the stability of the 5 selected reference genes. CL5272.Contig1_F/Unigene12410_D were consistently ranked as the most stably expressed in different tissues, at different developmental phases, and between two species, and were thus used as the reference genes. Gene-specific primers (Supplementary Table 1) were designed using Primer Premier 5.0 (Lalitha, 2000) based on assembled sequences. Relative expression levels of target genes were calculated using the 2^–ΔΔ*Ct*^ method (Livak and Schmittgen, 2001).

### Full-Length cDNA Cloning, Sequencing, and Phylogenetic Analysis

To explore the divergence and phylogenetic relationships of the genes of interest, the full-length coding sequences of interested genes were cloned and sequenced. RT-PCR was conducted to clone and validate the sequences of genes with complete open reading frames (ORFs) assembled. Primers (Supplementary Table 1) were designed based on the predicted coding sequences and alignments against the GenBank database. The first-strand cDNA’s were synthesized using the PrimeScript RT (Perfect Real Time) Kit (Takara Bio). Purified PCR products were cloned into pMD^TM^ 18-T vector (Takara Bio) and sequenced. For partial coding unigenes, the RACE (Rapid Amplification of cDNA Ends) method was implemented using the SMARTer^®^ RACE Amplification Kit (Takara Bio). Gene-specific primers for the 5′- and 3′-RACE reactions were designed based on conserved regions identified from the multiple sequence alignments of different genes. Following cloning and sequencing the overlapping 5′ and 3′-RACE fragments, pairs of “end-to-end” primer combinations were designed and used to amplify the full-length cDNA. The amplified products were then purified and cloned into pMD^TM^ 18-T vector for sequencing. Sequence identities were confirmed by BLAST searches against the GenBank database. The gene sequences obtained in this study have been submitted to the GenBank database with the accession codes: MH748779-MH748792. Phylogenetic analyses were carried out using the neighbor-joining method implemented in MEGA (Tamura et al., 2007).

### Construction and Comparison of Gene Co-expression Networks

To identify genes potentially associated with the varied accumulation of unsaturated fatty acids in different species, network-based comparative analyses were carried out based on time course gene expression data. TransDecoder^7^ was used to predict the coding sequences (CDS) and the peptide sequences from the assembled transcripts. Cd-hit was used to cluster contigs sharing highly identical CDS (with identity of over 90% and coverage of over 80%) (Li and Godzik, 2006). Expression values of the members included in a cluster were summed up, and the longest transcript was retained as the representative sequence. The Reciprocal Best Hits (RBH) procedure was applied to identify one to one orthologous pairs between *P. ostii* and *P. ludlowii*, which allowed for a detailed cross-species comparison (Movahedi et al., 2012). The NOISeq R package was used to screen interspecifically differentially expressed genes (Tarazona et al., 2011). Gene co-expression networks were constructed using the WGCNA R software package (Langfelder and Horvath, 2008). The matrices of pairwise Pearson correlation coefficients between all pairs of transcripts were generated and transformed into adjacency matrices using the formula: adjacency value = |(1 + correlation)/2| ^β^. β represents the soft-thresholding power for the correlation matrix, which gives greater weight to strongest correlations while maintaining the gene-gene connectivity. The resulting adjacency matrices was then converted to topological overlap (TO) matrices *via* TOM similarity algorithm, and genes were hierarchically clustered based on TO similarity. The dynamic tree-cutting algorithm was used to cut the hierarchal clustering dendrogram, and modules were defined as branches from the tree cutting. The modules containing genes encoding core fatty acid synthesis enzymes and showing similar expression patterns were selected and merged to generate the oil biosynthesis module. The robustness of the merged module was validated using 5,000 permutation tests (*P* value < 10^–3^; Zhan et al., 2015). The hyper-geometric test was performed for validating the selected gene set associated with oil biosynthesis (*P* value < 10^–6^). The program Cytoscape was used to visualize the network (Shannon et al., 2003).

To confirm the potential roles of predicted genes in promoting polyunsaturated fatty acid synthesis and accumulation, the expression data of seed development of two other ALA-rich plants, *C. sativa* and *P. frutescens*, were integrated into analysis (Kagale et al., 2016; Kim et al., 2016). The PhyloMCL algorithm was used to sort the predicted peptide sequences of different species into groups of putative orthologs on the basis of sequence similarity with default parameters (Zhou et al., 2020). Gene co-expression networks were constructed and analyzed using the aforementioned methods.

## Results

### Accumulation Dynamics of FA’s in Developing Seeds

**FIGURE 1:**
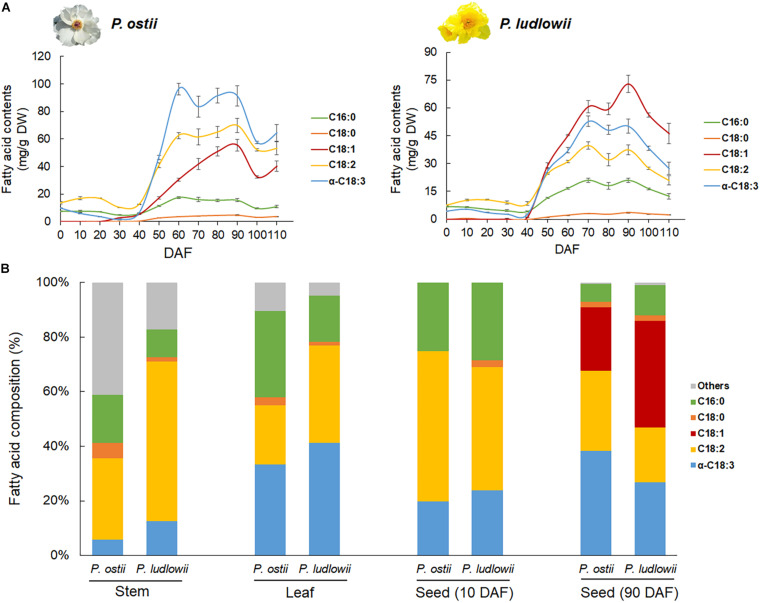
Fatty acid content and composition of the seed oils of *P. ostii* and *P. ludlowii*. **(A)** Accumulation dynamics of fatty acids during seed development, showing the similar trends of fatty acid accumulation but different fatty acid proportions between two species; **(B)** Relative proportions of various fatty acids in different tissues, showing inter-tissue variations in the fatty acid composition. The long chain SFA’s including arachidic acid (C20:0) and behenic acid (C22:0) were grouped into a single category (Others). Quoted values are means ± SE (*N* = 3). DAF: days after flowering.

*Paeonia ostii* and *P. ludlowii* exhibited a similar trend of change in oil content, though the total oil contents in mature seeds were a bit different between species (Supplementary Figure 1). At the early stage of seed development (0∼40 DAF), the oil content remained constant and low in both species. A critical transition occurred at around 40 DAF, with a sharp increase in oil content that peaked at 90 DAF and dropped slightly afterward (Figure 1A). Developmental stage-specific changes were detected not only in oil contents but also in oil compositions. Fatty acid profiling revealed five predominant components of the tree peony seed oil, i.e., stearic acid (SA), palmitic acid (PA), OA, LA, and ALA. The profiles of FA composition did not show significant difference between species at the early stages of seed development. Interspecific changes in FA composition emerged along with the massive accumulation of oil starting from 40 DAF to seed maturation. The major difference lay in the relative proportions of mono- and polyunsaturated fatty acids in the oils of different species. ALA was the most abundant fatty acid in the oil of *P. ostii* followed by LA and OA, whereas in the oil of *P. ludlowii*, OA was the predominant fatty acid (Figure 1A). Compared with that of *P. ludlowii*, the oil of *P. ostii* had a higher degree of fatty acid unsaturation. Interestingly, this pattern was reversed in stem and leaf tissues. Comparing the fatty acid profiles of mature seeds with those derived from stem and leaf tissues of *P. ostii* and *P. ludlowii* revealed that *P. ludlowii* contained a remarkably higher proportion of PUFA (LA and ALA) than *P. ostii* in stems and leaves (Figure 1B). The results also showed that long chain SFA’s including arachidic acid (C20:0) and behenic acid (C22:0) were relatively abundant in stem tissues, leading to a higher ratio of SFA:UFA in stems. By contrast, the high concentration of OA in mature seeds increased the ratios of MUFA:PUFA in seed oils, especially in *P. ludlowii*. Calculating the product-substrate ratio revealed a relatively lower omega-6 desaturation efficiency in *P. ludlowii* than in *P. ostii*, but not for omega-3 desaturation reaction (Supplementary Figure 2).

### *De novo* Transcriptome Assembly and Time-Course Gene Expression Profiling

*De novo* transcriptome assembly using 73.71 million and 73.63 million clean reads generated 54,655 and 57,665 unigenes in *P. ostii* and *P. ludlowii*, respectively, with an average length of 911 and 868 nt, N50 length of 1,447 and 1,328 nt, and overall annotation rate of 63.59 and 59.31% (Supplementary Table 2). The analogous distribution patterns of categorized annotations suggested that the expressed genes of two species were involved in similar functional categories and metabolic pathways (Supplementary Figure 3). The results of BLAST searches against the *Arabidopsis* Lipid Gene Database^8^ revealed 819 and 759 unigenes in *P. ostii* and *P. ludlowii* respectively, to be associated with lipid metabolism and signaling.

A total of 30 independent cDNA libraries were generated and sequenced for evaluating temporal gene expression patterns during seed development in *P. ostii* and *P. ludlowii*. Over 20 million clean reads were generated for each library. The clean reads were then aligned respectively, to *P. ostii* and *P. ludlowii* reference transcriptomes assembled. More than 90% of the reads were successfully aligned to the reference transcriptomes, with the least mapped sample showing a ratio of 86.11% (Supplementary Table 3).

A total of 8,089 and 14,218 unigenes identified in *P. ostii* and *P. ludlowii*, respectively, were differentially expressed at different seed development stages. Hierarchical clustering of differentially expressed genes (DEGs) generated ten clusters of DEG with similar expression dynamics in each species (Supplementary Figure 4). The unigenes included in each cluster were listed in Supplementary Tables 4, 5. Of them, the unigenes included in the cluster1, 4, and 5 of *P. ostii* and those from the cluster1, 4, and 7 of *P. ludlowii* showed bell-shaped expression patterns in agreement with the seed oil accumulation dynamics in both species. Functional enrichment analyses indicated that these genes were majorly involved in starch and sucrose metabolism, ALA metabolism, LA metabolism, biosynthesis of secondary metabolites, etc., (Supplementary Table 6).

### Cross-Species Comparison of Expression Stoichiometry of Oil Biosynthesis Pathway Genes

**FIGURE 2:**
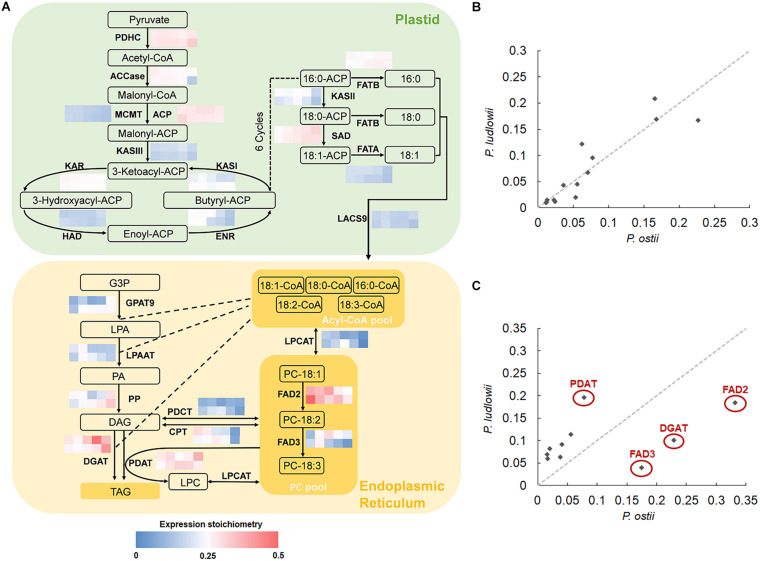
Cross-species comparison of expression stoichiometry of genes involved in fatty acid synthesis (FAS) and triacylglycerol (TAG) assembly. **(A)** Relative transcript abundance of genes encoding the core enzymes (indicated in bold) involved in lipid synthesis, with the grid showing the ratios of transcripts of each gene to the sum of FAS and TAG assembly transcripts at different developmental stages (0, 40, 50, 60, and 100 DAF from left to right) in *P. ostii* (upper) and *P*. *ludlowii* (lower), respectively; **(B)** Correlations of the expression stoichiometries of the 14 enzymes involved in *de novo* fatty acid synthesis at 50 DAF between *P. ostii* and *P. ludlowii*; **(C)** Correlations of the expression stoichiometries of the 10 enzymes involved in TAG assembly at 50 DAF between two species. Abbreviations are as follows: PDHC, plastidial pyruvate dehydrogenase complex; ACC, acetyl-CoA carboxylase; CoA, coenzyme A; ACP, acyl carrier protein; MCMT, malonyl-CoA ACP transferase; KAS, ketoacyl-ACP synthase; KAR, 3-ketoacyl-ACP reductase; HAD, 3-hydroxyacyl-ACP dehydratase; ENR, 2-enoyl-ACP reductase; FATA, acyl-ACP thioesterase A; FATB, acyl-ACP thioesterase B; SAD, stearoyl-ACP desaturases; LACS, long-chain acyl-CoA synthetase; FAD2, fatty acid desaturase 2; FAD3, fatty acid desaturase 3; GPAT9, glycerol 3-phosphate acyltransferase 9; LPAAT, lysophosphatidic acid acyltransferase; LPCAT, lysophosphatidylcholine acyltransferase; DGAT, acyl-CoA: diacylglycerol acyltransferase; PDAT, phospholipid: diacylglycerol acyltransferase; CPT, CDP-choline: DAG cholinephosphotransferase; PDCT, phosphatidylcholine:diacylglycerol cholinephosphotransferase; G3P, glycerol-3-phosphate; LPA, lysophosphatidic acid; PA, phosphatidic acid; LPC, lysophosphatidylcholine; PC, phosphatidylcholine; DAG, diacylglycerol; TAG, triacylglycerol.

The key biological processes involved in oil synthesis have been well studied in plants: FA’s are exclusively synthesized in plastids and then the activated FA’s are exported to endoplasmic reticulum (ER) for modification and TAG assembly (Bates et al., 2013). Genes encoding the core enzymes involved in FA synthesis, modification and subsequent incorporation into TAG’s have been identified, which are conserved among plants (Troncoso-Ponce et al., 2011). It is thus possible to detect the potential rate-limiting steps and enzymes associated with interspecific variation in oil components by calculating and comparing the transcriptional abundance of genes encoding for the core enzymes based on seed-specific genome-wide expression data. The results showed that the genes involved in *de novo* FA synthesis in plastids displayed similar stoichiometries between species (Figure 2), and maintained strong correlations throughout seed development with *r*^2^ values ranging from 0.74 to 0.96, with an average inter-species expression stoichiometry CV value of 0.37. In contrast to the globally conserved expression stoichiometry of genes involved in *de novo* FA synthesis, genes associated with FA modification and TAG assembly underwent divergence in transcriptional abundance. Of them, the genes encoding FAD2, FAD3, DGAT (acyl-CoA: diacylglycerol acyltransferase), and PDAT (phospholipid: diacylglycerol acyltransferase) exhibited distinct stoichiometries in different species, with the CV values of stoichiometry of 0.49, 0.69, 0.65, and 0.41, respectively. The correlations of expression stoichiometries of various genes involved in TAG assembly were not statistically significant during oil-accumulation stages (Figure 2C).

### Sequence and Expression Validation of Distinctly Expressed Desaturase and Acyltransferase Genes

Based on assembled transcripts, the complete coding sequences of *FAD2* and *FAD3* were cloned and sequenced in both *P. ostii* and *P. ludlowii*. The expression levels of all desaturase and terminal acyltransferase genes throughout seed development were validated by RT-qPCR. The results revealed the existence of distinct transcripts for all of the genes examined. Sequence comparison and phylogenetic analysis showed that *P. ostii* and *P. ludlowii* had at least two *FAD2* and three *FAD3* paralogous genes, designated respectively, as *Po/PlFAD2-1*, *Po/PlFAD2-2*, *Po/PlFAD3-1*, *Po/PlFAD3-2*, and *Po/PlFAD3-3*. The tree peony *FAD2* paralogous genes formed two separate groups with those from other plants in the phylogenetic tree, suggesting that they were derived from a duplication event happened in the common ancestor of different plants (Supplementary Figure 5). Instead, the *FAD3* paralogs pairs from *P. ostii* and *P. ludlowii* clustered together in the phylogenetic tree, forming an independent clade with three paralogous branches (Supplementary Figure 6A). Two lineage-specific duplication events might have taken place subsequently prior to the divergence of tree peony species to produce the three *FAD3* paralogous genes. All tree peony *FAD3* genes contained three histidine rich motifs. The *FAD3-1* branch was distinct from the other two branches for lacking of the isoleucine to valine substitution in the third histidine rich motif (Supplementary Figure 6B).

**FIGURE 3:**
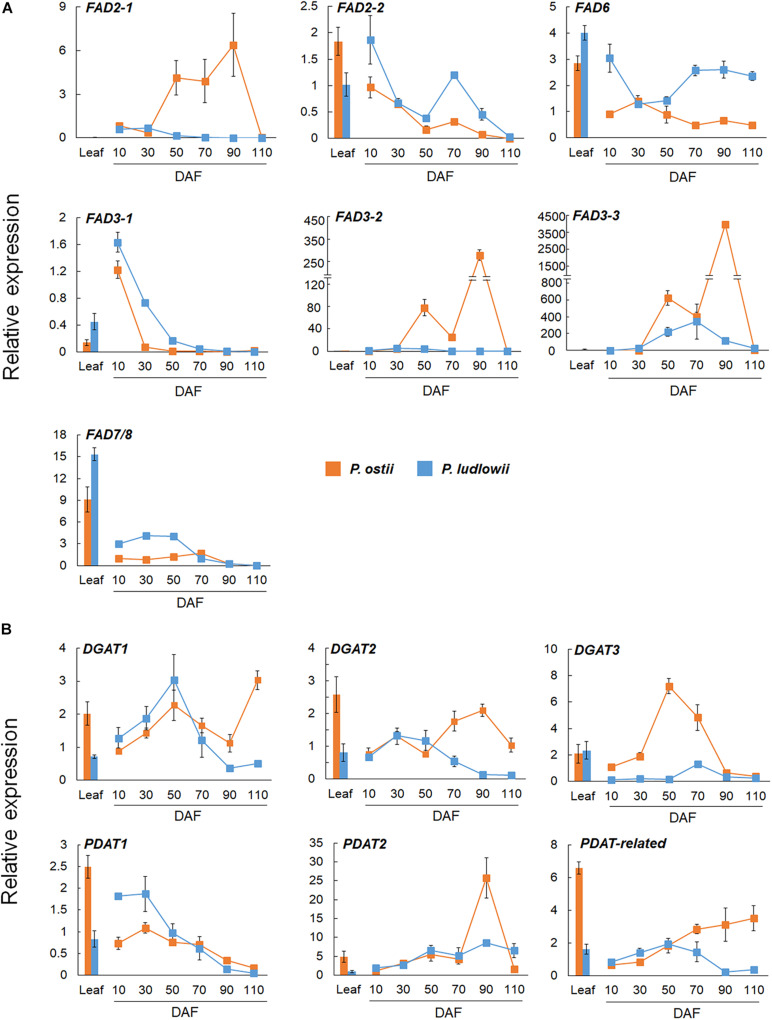
RT-qPCR validation of distinctly expressed desaturase and acyltransferase genes in *P. ostii and P. ludlowii*. **(A)** Differential expression patterns of the duplicates of *FAD2* and *FAD3* in different tissues and at different stages of seed development; **(B)** Differential expression patterns of analogous *DGAT* and paralogous *PDAT* genes in different tissues and at different stages of seed development. Relative expression values, normalized to the selected reference gene, were shown as 2^–ΔΔ*CT*^ relative to 10 DAF of *P. ostii*. Error bars represent the SE of three biological replicates with three technical replicates each.

A significant correlation was found between the results of RT-qPCR and RNA-Seq, as evaluated by linear regression analysis (*r*^2^ = 0.91; *p* < 0.0001; Supplementary Figure 7). Differential expressions of paralogous genes were detected in both *FAD2* and *FAD3* genes. As shown in Figure 3A, *FAD2-2* was highly expressed in leaves and young seeds, and was down-regulated at seed maturing stages. In contrast, *FAD2-1* was exclusively expressed in seeds. It was up regulated during seed maturation, and showed significantly higher expression levels in *P. ostii* than in *P. ludlowii*. Similarly, *FAD3-1* was mainly expressed in leaves and young seeds, while *FAD3-2* and *FAD3-3* were mainly expressed in maturing seeds. The expressions of *FAD3-2* and *FAD3-3* were evidently increased in the oil accumulating stages. The relative expression level of *FAD3-3* was much higher than *FAD3-2* in both species, and the total level of expression of *FAD3-2* and *FAD3-3* in *P. ostii* was significantly higher than that in *P. ludlowii*.

DGAT’s were a group of enzymes that play critical roles in TAG synthesis and accumulation. Previous studies have shown that DGAT’s were encoded by multiple genes that evolved independently but became functionally convergent during evolution (Liu et al., 2012). In this study, three analogous *DGAT* transcripts were identified in each species, *Po/PlDGAT1*, *Po/PlDGAT2*, and *Po/PlDGAT3*, which were homologous to *Arabidopsis* At2g19450, At3g51520, and At1g48300, respectively. Among them, *DGAT3* was mainly expressed during the oil-accumulating development stage, and showed a much higher expression level in *P. ostii* than in *P. ludlowii*. *DGAT1* and *DGAT2* were expressed in both leaves and seeds with a lower expression level compared to *DGAT3*. As shown in Figure 3B, the expression levels of *DGAT1* and *DGAT2* in *P. ostii* were lower than those in *P. ludlowii* during the early stages of seed development, but the situation reversed at the later stages. PDAT has been proposed to be involved in TAG biosynthesis *via* an acyl-CoA independent pathway, enhancing oil accumulation (Bates et al., 2013). The *PDAT* transcripts homologous to *Arabidopsis PDAT1* (At5g13640), *PDAT2* (At3g44830), and the *PDAT*-*related* gene (At4g19860) respectively, were identified in both *P. ostii* and *P. ludlowii*. The expression level of *PoPDAT2* in mature seeds was much higher than that of *PlPDAT2*. *PDAT1* was clearly down-regulated with progressing seed development, whereas the *PDAT*-*related* gene was continuously up-regulated in *P. ostii* but not in *P. ludlowii*.

### Network-Based Identification of Genes Potentially Associated With PUFA Biosynthesis and Accumulation

The gene co-expression network was constructed using WGCNA. A total of 23 co-expression modules were generated for *P. ostii*, consisting of 21,842 genes. Highly correlated modules were merged to generate the oil biosynthesis module, which contained 3,745 genes. Among the 3,745 co-expressed genes, 687 genes were differentially expressed between *P. ostii* and *P. ludlowii*, suggesting their probable involvement in the regulation of FA desaturation and promoted ALA accumulation in *P. ostii*.

**FIGURE 4:**
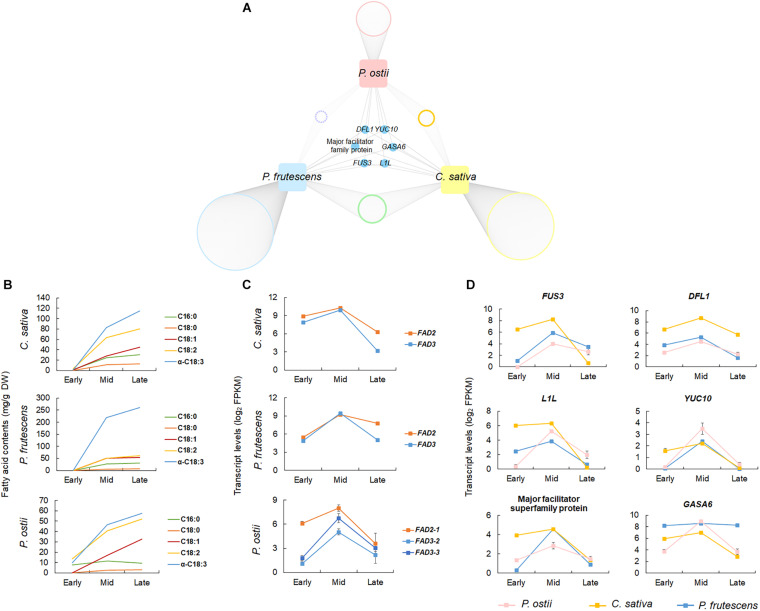
Comparative co-expression network analyses across *P. ostii*, *C. sativa*, and *P. frutescens*. **(A)** Network showing genes conservatively co-expressed with *FAD2* and *FAD3* in the three ALA-rich species; **(B)** Accumulation dynamics of fatty acids during seed development, showing the similar trends of fatty acid accumulation between three species; **(C)** Expression patterns of *FAD2* and *FAD3* at different stages of seed development in the three species; **(D)** Expression patterns of the conservatively co-expressed genes. Error bars indicate the SE of the mean.

To validate the potential regulatory roles of genes co-expressed with the oil biosynthesis pathway genes in *P. ostii*, the gene co-expression networks of two ALA-rich species *C. sativa* and *P. frutescens* were also constructed based on the gene expression profiles during seed development. The co-expression networks comprised 21,294 genes in *C. sativa*, and 24,754 in *P. frutescens*. The oil biosynthesis modules of *C. sativa* and *P. frutescens* consisted of 4,454 and 2,329 genes, respectively. Comparing the co-expressed gene sets with that of *P. ostii* revealed 265 orthogroups that were shared by three ALA-rich species. A total of 362 genes co-expressed in *P. ostii* were also co-expressed in other two species. Of them, 91 were differentially expressed between *P. ostii* and *P. ludlowii* (Supplementary Table 7). Go enrichment analysis revealed that these genes were majorly involved in transcriptional regulation (such as genes encoding the oil store regulating genes *WRI1*, the basic helix-loop-helix proteins *bHLH113*, and the ethylene responsive factor *ERF72*), protein modification (such as the protein kinase genes and the ubiquitin-conjugating enzyme gene *UBC5*), signal transduction (such as *JAZ1* involved in the jasmonic acid mediated signaling pathway), and metabolic processes (Supplementary Table 8). A few genes were found to be conservatively co-expressed with *FAD2* and *FAD3* across three ALA-rich species (Figure 4 and Supplementary Figure 8). Apart from some genes previously known to regulate desaturase genes, such as FUSCA3 (*FUS3*) and LEAFY COTYLEDON1-like (*L1L*), these genes included genes encoding the flavin-containing monooxygenase family protein (*YUC10*), the auxin-responsive GH3 family protein (*DFL1*), gibberellin-regulated family protein (*GASA6*), and genes encoding the proteins from the major facilitator superfamily. Few genes were detected to be conservatively co-expressed with *DGAT3* and *PDAT2* in three ALA-high species.

## Discussion

Given the indispensability of PUFA for human health and the distinct properties required for industrial uses, it is of great necessity to decipher key regulators and possible mechanisms underlying variations in seed oil fatty acid composition (Dyer et al., 2008). *P. ostii* and *P. ludlowii* are congeneric species but have diverged in FA accumulation patterns, with ALA and OA being the most abundant fatty acids in *P. ostii* and *P. ludlowii* seed oils, respectively. The higher percentage of polyunsaturated fatty acid indicated a greater degree of fatty acid unsaturation in the *P. ostii* seed oil. However, within stems and leaves, the levels of LA and ALA in *P. ostii* were lower than in *P. ludlowii*. It was also found that the abundance of OA remained very low in stems and leaves, which could hardly be detected in both species. These results not only suggested that the differences in seed oil composition between *P. ostii* and *P. ludlowii* were unlikely to be plastic changes induced by environmental variation since previous studies have shown increased proportions of unsaturated fatty acids in plants at lower temperatures/higher altitudes (Linder, 2000; Zhang et al., 2015; Guerin et al., 2020), but also indicated that the reactions of fatty acid desaturation were controlled by distinct regulatory mechanisms in different organs.

The relative proportions of unsaturated FA’s in seed oils were largely determined by the stepwise desaturation processes in the oil biosynthesis pathway. The expression levels of individual FAD’s and their specificity and efficiency in catalyzing fatty acid desaturation play a key role in controlling the component ratios and the unsaturation level of seed oils. The *FAD2* and *FAD3* genes of tree peony have experienced gene duplication events during evolution. The varied expression patterns of the *FAD2* and *FAD3* duplicates in different organs clearly showed subfunctionaliztion of *FAD2* and *FAD3* paralogs following duplication. The significantly higher expressions of *FAD2-1*, *FAD3-2*, and *FAD3-3* in *P. ostii* during seed maturing period were consistent with the high proportion of PUFA’s in the *P. ostii* seed oil. Instead, the downregulation of *FAD2-1* in the maturing seeds of *P. ludlowii* probably induced reduction in omega-6 desaturation efficiency, decreasing the conversion rate of OA to LA and consequently leading to the higher accumulation of OA in *P. ludlowii*.

The last step in TAG biosynthesis was catalyzed by two types of enzymes: DGAT and PDAT. DGAT catalyzed the acyl-CoA-dependent biosynthesis of TAG, which has been proposed to be a bottleneck for oil accumulation in some plant species (Cahoon et al., 2007). Different types of DGAT’s with similar catalytic properties and no sequence homology have been identified (Liu et al., 2012; Bates et al., 2013). Gene expression profiling revealed that the tree peony *DGAT* genes homologous to *Arabidopsis DGAT1*, *DGAT2* and *DGAT3*, respectively, were differentially expressed at different stages of seed development and between two peony species. The expression profiles of different genes shared a common feature that the expression levels of each gene in *P. ostii* were significantly higher than in *P. ludlowii* from mid to late stages of seed development (50-110 DAF). *DGAT3* showed the most significant difference between species. Previous studies have shown that DGAT3 was distinct from DGAT1 and DGAT2 for its cytoplasmic localization due to lack of transmembrane domains. Although the soluble DGAT3 has been reported to participate in the cytosolic pathway of TAG synthesis, its specific role in TAG synthesis remains unclear. As demonstrated in peanut, soybean and *Arabidopsis* (Saha et al., 2006; Hernandez et al., 2012; Turchetto-Zolet et al., 2016), the putative *DGAT3* homologs in peony species were ubiquitously expressed, with higher expression levels in maturing seeds. The main difference lay in the most abundant accumulation of the *DGAT3-related* transcripts occurring at the mid but not the late stages of seed development. The correlations between the expression levels of *DGAT3* and the distinct fatty acid profiles observed in different peony species were yet to be clarified. Unlike DGAT’s, PDAT catalyzed the acyl-CoA-independent formation of TAG in plants. Previous studies on ALA-rich species, such as *Linum usitatissimum* and *Perilla frutescens*, have suggested the activity of PDAT to preferentially transfer ALA into TAG (Pan et al., 2013; Kim et al., 2016). Thus, compared to DGAT, PDAT might contribute more to TAG synthesis in seeds that were high in polyunsaturated fatty acids. The increased expression of peony *PDAT2* and the *PDAT-related* gene in *P. ostii* seemed to support this issue, though functional and expression divergence of *PDAT* paralogs had been detected in this study and in other plants (Pan et al., 2013, 2015; Zang et al., 2019).

The congruent patterns of variation at the phenotypic and molecular levels suggested that the relative transcriptional abundances of the enzymes involved in FA desaturation and TAG assembly were correlated with the differential accumulation of unsaturated FA’s in different peony species. The higher expression of *FAD2-1*, *FAD3-2*, and *FAD3-3* possibly promoted sequential FA desaturation in *P. ostii*, pushing the reaction toward the formation of PUFA. The functional relationships between the raised expression of *DGAT2, DGAT3*, and *PDAT2* and the preferential accumulation of ALA in *P. ostii* remain to be elucidated. Previous studies have shown the ALA-preference of a few DGAT and PDAT enzymes in other plants (Pan et al., 2013; Kim et al., 2016; Xu et al., 2018). Experimental evaluation of the selectivity of DGAT’s and PDAT’s in peony species will be helpful to clarify whether the enhanced expression of *DGAT2*, *DGAT3*, and *PDAT2* have contributed to the enrichment of ALA in *P. ostii* seed oil by preferentially transferring ALA into TAG. It was evident that the alterations in the relative proportions of the component fatty acids in seed oils and the developmental stage-specific changes in fatty-acid concentrations were not dependent solely on the activity of one single gene encoding enzyme, but on the synergistic effects of all pathway genes. Not only was the mRNA level of each gene encoding enzyme under the transcriptional control during seed development, but there could be some regulators participated in the spatial-temporal co-regulation of different genes and the coordination of upstream and downstream reaction modules. Deciphering the components that coordinately regulated the expression of multiple lipid biosynthetic genes and enzyme activity was essential for dissecting the regulatory mechanisms underlying variations in FA content and composition in seed oils.

Genes with coordinated expression were usually assumed to work cooperatively in related pathways, sharing similar function and regulation, especially when the co-expression relationships were conserved across species (Su et al., 2011; Medema and Osbourn, 2016). Gene co-expression analysis has been increasingly used to prioritize genes that might underlie a phenotype. In this study, we screened co-expressed gene pairs conserved across evolutionarily distant species and identified genes that were conservatively co-expressed with genes known to be involved in FA synthesis and desaturation. The results clearly showed that some of the co-expressed genes were well known as key regulators of seed oil generation, such as the transcription factor *WRI1*, which was identified to govern the flux of carbon through glycolysis and fatty acid synthesis by regulating genes associated with fatty acid biosynthesis in plastids (Cernac and Benning, 2004; Baud et al., 2007). The higher expression level of *WRI1* of *P. ostii* than that of *P. ludlowii* was also in accordance with the slightly higher oil content of *P. ostii*.

In addition to *WRI1*, a dozen other transcription factors of different families were also tightly co-expressed with the oil biosynthesis pathway genes, including the basic helix-loop-helix proteins (*bHLH113*), the ethylene responsive factors (*ERF72*), the MYB transcription factors (*MYB61, MYB86*) and the NAC transcription factors (*NAC100*), the B3 protein family (*HSL1*). Most of them or their homologs have been found to be associated with diverse aspects of lipid metabolism, though very few of them have been shown to modify fatty acid proportions. For instance, *bHLH113* has been shown to be up-regulated at different stages of mesocarp development to produce higher oil yield in oil palm and to be co-expressed with major lipid genes in *Hiptage benghalensis* (Wong et al., 2017; Tian et al., 2019). The role of hormones in regulating seed development and storage compound accumulation has been widely recognized (Yeap et al., 2017; Grimberg et al., 2018). Mutations in hormone-related transcription factors could also induce changes in the fatty acid profiles of seed oil, showing varied SFA: UFA (Hughes et al., 2008) and MUFA:PUFA ratios (Geilen et al., 2017). The functional relationships of the NAC and MYB transcription factors identified in this study to the high-level accumulation of ALA in seed oils remained largely unknown, without empirical evidences showing how they were connected. Nevertheless, such connections were supported by the genome-wide association study of *A. thaliana* for identifying determinants of variation in seed oil composition, which detected a group of MYB genes (*MYB10*, *MYB64*, *MYB67*, and *MYB103*) linked to the SNP’s associated with variation in fatty acid proportions (Branham et al., 2015).

Some genes involved in protein post-translational modification, such as protein phosphorylation (Protein kinase superfamily protein) and ubiquitination (*UBC5*), were also tightly co-expressed with fatty acid biosynthesis genes. Previous studies have shown that a serine/threonine/tyrosine protein kinase participated in the regulation of the content and composition of the *Arabidopsis* seed oil by mediating phosphorylation of oil body proteins (Ramachandiran et al., 2018). It has also been revealed that the seed-expressed casein kinase I enhanced the *bHLH*-mediated transactivation of the *FAD2* gene promoter *via* phosphorylation of the *bHLH* transcription factor (Kim et al., 2010), while the ubiquitin proteasome pathway modulated FAD3 protein amounts in response to temperature (O’Quin et al., 2010). The results of this study were thus consistent with previous studies that suggested that post-translational regulatory mechanisms played an important role in modulating FAD2 and FAD3 protein abundance and stability (Martz et al., 2006; Bourassa, 2008).

Network-based comparative transcriptome analysis also revealed genes with diverse functions to be conservatively co-expressed with *FAD2* and *FAD3* across different species. Some of them (*FUS3* and *L1L*) were previously known to participate in the regulation of *FAD3 via* (bZIP67:L1L:NF-YC2) transcriptional complex, whose induction was triggered by *FUS3* (Mendes et al., 2013). Though *bZIP67* was only found to be co-expressed with *FAD2* and *FAD3* in *C. sativa*, another group A *bZIP* member, *AREB3*, which was closed related to *bZIP67* was found to be co-expressed with *FAD2* and *FAD3* in *P. frutescens* and *P. ostii*, and was differentially expressed between *P. ostii* and *P. ludlowii*, previous research has also identified that *AREB3* was able to enhance *FAD3* expression in the presence of *L1L* and *NF-YC2* (Mendes et al., 2013). In *P. ostii*, *NF-YC2* gene was also found to be co-expressed with *FAD3* and *FAD2* while in *P. frutescens* and *C. sativa*, some other *NF-YC* family members were found to exhibit similar expression patterns with *FAD2* and *FAD3*, suggesting the potential role of other *NF-YC* family members in regulating desaturase genes. Apart from them, other co-expressed genes have not yet been reported to date to play roles in regulating fatty acid proportions in plants. Of them, *YUC10* (Jia et al., 2014), *DFL1* (Nemhauser et al., 2006) and *GASA6* (Qu et al., 2016) were associated with hormone biosynthesis or response. The function of the major facilitator superfamily proteins remained unclear in plants, though it has been shown that the major facilitator superfamily domain-containing protein was an omega-3 fatty acid transporter in human (Zhang W. et al., 2018; Zhou et al., 2019). However, their relationships with the variation of fatty acid composition were not only demonstrated by the conserved co-expression patterns in different plants shown in this study, but were also supported by early genome-wide association studies (Menard et al., 2017; Zhao et al., 2019). The roles of these genes in regulating fatty acid composition remained to be functionally characterized in future research.

## Conclusion

Fatty acid synthesis and desaturation involved a coordinated series of enzymatic reactions. Lineage-specific duplication and subsequent expression alterations of desaturase genes obviously contributed to the phenotypic divergence in the relative proportions of unsaturated fatty acids in the seed oils of peony species. However, the variation in fatty acid proportions was not entirely dependent on desaturase genes. A few genes associated with transcriptional and translational control (such as *NAC100*, *MYB61*, and *UBC5*) and hormonal regulation (such as *YUC10*, *DFL1*, and *GASA6*) seemed to be also involved to regulate the desaturase activity and coordinate various enzymatic steps. These genes were highly correlated with phenotypic variation, and were tightly co-expressed with desaturase genes, providing new targets for future functional characterization and bioengineering to extend our understanding of the regulatory mechanisms underpinning variations in fatty acid composition and unsaturation levels, and to help improve the composition of nutritional and industrial oils.

## Data Availability Statement

The datasets presented in this study can be found in online repositories. The names of the repository/repositories and accession number(s) can be found in the article/Supplementary Material.

## Author Contributions

MW performed the wet lab work and data analysis and drafted the manuscript. GL, CZ, and JJ helped to set up experiments. ZX helped the material collection. YW, WZ, and ZS participated in the data analysis. LG, YH, and JY conceived the idea, participated in the design of the study, and finalized the manuscript. All of the authors read and approved the final manuscript.

## Conflict of Interest

The authors declare that the research was conducted in the absence of any commercial or financial relationships that could be construed as a potential conflict of interest.

## Footnotes

1 http://www.ncbi.nlm.nih.gov/sra

2 http://www.ncbi.nlm.nih.gov

3 http://www.ebi.ac.uk/uniprot/

4 http://www.arabidopsis.org

5 http://www.geneontology.org

6 http://www.genome.jp/kegg

7 http://transdecoder.sourceforge.net/

8 http://aralip.plantbiology.msu.edu/

## References

[B1] Andersen C. L. Jensen J. L. Ørntoft T. F. (2004). Normalization of real-time quantitative reverse transcription-PCR data: a model-based variance estimation approach to identify genes suited for normalization, applied to bladder and colon cancer data sets. *Cancer Res.* 64 5245–5250. 10.1158/0008-5472.CAN-04-0496 15289330

[B2] Bates P. D. Stymne S. Ohlrogge J. Browse J. Farmer E. (2013). Biochemical pathways in seed oil synthesis. *Curr. Opin. Plant Biol.* 16 358–364. 10.1016/j.pbi.2013.02.015 23529069

[B3] Baud S. Mendoza M. S. To A. Harscoët E. Lepiniec L. Dubreucq B. (2007). WRINKLED1 specifies the regulatory action of LEAFY COTYLEDON2 towards fatty acid metabolism during seed maturation in *Arabidopsis*. *Plant J.* 50 825–838. 10.1111/j.1365-313X.2007.03092.x 17419836

[B4] Bourassa L. (2008). *Post-translational regulation of plant fatty acid desaturases as expressed in Saccharomyces cerevisiae.* New Orleans, LA: University of New Orleans [master’s thesis].

[B5] Branham S. E. Wright S. J. Reba A. Linder C. R. (2015). Genome-Wide association study of *Arabidopsis thaliana* identifies determinants of natural variation in seed oil composition. *J. Hered.* 107 248–256. 10.1093/jhered/esv100 26704140 PMC4885229

[B6] Cahoon E. B. Shockey J. M. Dietrich C. R. Gidda S. K. Mullen R. T. Dyer J. M. (2007). Engineering oilseeds for sustainable production of industrial and nutritional feedstocks: solving bottlenecks in fatty acid flux. *Curr. Opin. Plant Biol.* 10 236–244. 10.1016/j.pbi.2007.04.005 17434788

[B7] Cao S. Zhou X. Wood C. Green A. G. Singh S. P. Liu L. (2013). A large and functionally diverse family of *Fad2* genes in safflower (*Carthamus tinctorius* L.). *BMC Plant Biol.* 13:5. 10.1186/1471-2229-13-15 23289946 PMC3554562

[B8] Cernac A. Benning C. (2004). WRINKLED1 encodes an AP2/EREB domain protein involved in the control of storage compound biosynthesis in *Arabidopsis*. *Plant J.* 40 575–585. 10.1111/j.1365-313X.2004.02235.x 15500472

[B9] Chen Y. Meesapyodsuk D. Qiu X. (2014). Transgenic production of omega-3 very long chain polyunsaturated fatty acids in plants: accomplishment and challenge. *Biocatal. Agric. Biotechnol.* 3 38–43. 10.1016/j.bcab.2013.08.007

[B10] Conesa A. Gotz S. Garciagomez J. M. Terol J. Talon M. Robles M. (2005). Blast2GO: a universal tool for annotation, visualization and analysis in functional genomics research. *Bioinformatics* 21 3674–3676. 10.1093/bioinformatics/bti610 16081474

[B11] Damude H. G. Kinney A. J. (2008). Enhancing plant seed oils for human nutrition. *Plant Physiol.* 147 962–968. 10.1104/pp.108.121681 18612073 PMC2442541

[B12] Durrett T. P. Benning C. Ohlrogge J. (2008). Plant triacylglycerols as feedstocks for the production of biofuels. *Plant J.* 54 593–607. 10.1111/j.1365-313X.2008.03442.x 18476866

[B13] Dyer J. M. Stymne S. Green A. G. Carlsson A. S. (2008). High−value oils from plants. *Plant J.* 54 640–655. 10.1111/j.1365-313X.2008.03430.x 18476869

[B14] Geilen K. Heilmann M. Hillmer S. Böhmer M. (2017). WRKY43 regulates polyunsaturated fatty acid content and seed germination under unfavourable growth conditions. *Sci. Rep.* 7:14235 10.1038/s41598-017-14695-14690 PMC5660175 29079824

[B15] Grabherr M. Haas B. J. Yassour M. Levin J. Z. Thompson D. A. Amit I. (2011). Full-length transcriptome assembly from RNA-Seq data without a reference genome. *Nat. Biotechnol.* 29 644–652. 10.1038/nbt.1883 21572440 PMC3571712

[B16] Grimberg Å Lager I. Street N. R. Robinson K. M. Marttila S. Mähler N. (2018). Storage lipid accumulation is controlled by photoperiodic signal acting via regulators of growth cessation and dormancy in hybrid aspen. *New Phytol.* 219 619–630. 10.1111/nph.15197 29761498

[B17] Guerin C. Serret J. Montúfar R. Vaissayre V. Bastos-Siqueira A. Durand-Gasselin T. (2020). Palm seed and fruit lipid composition: phylogenetic and ecological perspectives. *Ann. Bot.* 125 157–172. 10.1093/aob/mcz175 31665224 PMC7080222

[B18] Han J. Liu Z. Li X. Li J. Hu Y. (2016). Diversity in seed oil content and fatty acid composition in three tree peony species with potential as sources of omega-3 fatty acids. *J. Hortic. Sci. Bi.* 91 175–179. 10.1080/14620316.2015.1133538

[B19] Han Y. Xu G. Du H. Hu J. Liu Z. Li H. (2017). Natural variations in stearoyl-acp desaturase genes affect the conversion of stearic to oleic acid in maize kernel. *Theor. Appl. Genet.* 130 151–161. 10.1007/s00122-016-2800-2805 27717956

[B20] Haslam R. P. Ruiz-Lopez N. Eastmond P. Moloney M. Sayanova O. Napier J. A. (2013). The modification of plant oil composition via metabolic engineering—better nutrition by design. *Plant Biotechnol. J.* 11 157–168. 10.1111/pbi.12012 23066823

[B21] Hernandez M. L. Whitehead L. F. He Z. Gazda V. Gilday A. D. Kozhevnikova E. (2012). A cytosolic acyltransferase contributes to triacylglycerol synthesis in sucrose-rescued *Arabidopsis* seed oil catabolism mutants. *Plant Physiol.* 160 215–225. 10.1104/pp.112.201541 22760209 PMC3440200

[B22] Hughes R. Spielman M. Schruff M. C. Larson T. R. Graham I. A. Scott R. J. (2008). Yield assessment of integument−led seed growth following targeted repair of auxin response factor 2. *Plant Biotechnol. J.* 6 758–769. 10.1111/j.1467-7652.2008.00359.x 18643948

[B23] Ihaka R. Gentleman R. (1996). R: a language for data analysis and graphics. *J. Comput. Graph. Stat.* 5 299–314. 10.1080/10618600.1996.10474713

[B24] Jia H. Suzuki M. Mccarty D. R. (2014). Regulation of the seed to seedling developmental phase transition by the LAFL and VAL transcription factor networks. *Wiley Interdiscip. Rev. Dev. Biol.* 3 135–145. 10.1002/wdev.126 24902838 PMC4282589

[B25] Kagale S. Nixon J. Khedikar Y. Pasha A. Provart N. J. Clarke W. E. (2016). The developmental transcriptome atlas of the biofuel crop *Camelina sativa*. *Plant J.* 88:879. 10.1111/tpj.13302 27513981

[B26] Kim H. U. Lee K. R. Shim D. Lee J. H. Chen G. Q. Hwang S. (2016). Transcriptome analysis and identification of genes associated with ω-3 fatty acid biosynthesis in *Perilla frutescens* (L.) var. *frutescens*. *BMC Genomics* 17:474 10.1186/s12864-016-2805-2800 PMC4920993 27342315

[B27] Kim M. J. Go Y. S. Lee S. B. Kim Y. S. Shin J. S. Min M. K. (2010). Seed-expressed casein kinase I acts as a positive regulator of the *SeFAD2* promoter via phosphorylation of the *SebHLH* transcription factor. *Plant Mol. Biol. Report.* 73 425–437. 10.1007/s11103-010-9630-9637 20349267

[B28] Kodali D. R. (2002). High performance ester lubricants from natural oils. *Ind. Lubr. Tribol.* 54 165–170. 10.1108/00368790210431718

[B29] Lalitha S. (2000). Primer premier 5. *Biotech. Softw. Internet. Rep.* 1 270–272. 10.1089/152791600459894

[B30] Langfelder P. Horvath S. (2008). WGCNA: an R package for weighted correlation network analysis. *BMC Bioinform.* 9:559. 10.1186/1471-2105-9-559 19114008 PMC2631488

[B31] Li B. Dewey C. N. (2011). RSEM: accurate transcript quantification from RNA-Seq data with or without a reference genome. *BMC Bioinform.* 12:323. 10.1186/1471-2105-12-323 21816040 PMC3163565

[B32] Li H. Peng Z. Yang X. Wang W. Fu J. Wang J. (2013). Genome-wide association study dissects the genetic architecture of oil biosynthesis in maize kernels. *Nat. Genet.* 45 43–72. 10.1038/ng.2484 23242369

[B33] Li S. S. Wang L. S. Shu Q. Y. Wu J. Chen L. G. Shao S. (2015). Fatty acid composition of developing tree peony (*Paeonia* section *Moutan* DC.) seeds and transcriptome analysis during seed development. *BMC Genomics* 16:208 10.1186/s12864-015-1429-1420 PMC4404109 25887415

[B34] Li S. Yuan R. Chen L. Wang L. Hao X. Wang L. (2015). Systematic qualitative and quantitative assessment of fatty acids in the seeds of 60 tree peony (*Paeonia* section *Moutan* DC.) cultivars by GC-MS. *Food Chem.* 173 133–140. 10.1016/j.foodchem.2014.10.017 25466004

[B35] Li W. Godzik A. (2006). Cd-hit: a fast program for clustering and comparing large sets of protein or nucleotide sequences. *Bioinformatics* 22:1658. 10.1093/bioinformatics/btl158 16731699

[B36] Linder C. R. (2000). Adaptive evolution of seed oils in plants: accounting for the biogeographic distribution of saturated and unsaturated fatty acids in seed oils. *Am. Nat.* 156 442–458. 10.1086/303399 29592140

[B37] Liu H. Gao L. Hu Y. (2015). Reference genes discovery and selection for quantitative real-time PCR in tree peony seed and petal tissue of different development stages. *J. Agric. Biotechnol.* 23 1639–1648. 10.3969/j.issn.1674-7968.2015.12.014

[B38] Liu Q. Siloto R. M. Lehner R. Stone S. J. Weselake R. J. (2012). Acyl-CoA:diacylglycerol acyltransferase: molecular biology, biochemistry and biotechnology. *Prog. Lipid Res.* 51:350. 10.1016/j.plipres.2012.06.001 22705711

[B39] Livak K. J. Schmittgen T. D. (2001). Analysis of relative gene expression data using real-time quantitative PCR and the 2^−ΔΔCT^ Method. *Methods* 25 402–408. 10.1006/meth.2001.1262 11846609

[B40] Martz F. Kiviniemi S. Palva T. E. Sutinen M.-L. (2006). Contribution of omega-3 fatty acid desaturase and 3-ketoacyl-ACP synthase II (KASII) genes in the modulation of glycerolipid fatty acid composition during cold acclimation in birch leaves. *J. Exp. Bot.* 57 897–909. 10.1093/jxb/erj075 16473891

[B41] Medema M. H. Osbourn A. (2016). Computational genomic identification and functional reconstitution of plant natural product biosynthetic pathways. *Nat. Prod. Rep.* 33 951–962. 10.1039/C6NP00035E 27321668 PMC4987707

[B42] Menard G. Martin M. J. Bryant F. Munoz-Azcarate O. Kelly A. A. Hassani-Pak K. (2017). Genome wide analysis of fatty acid desaturation and its response to temperature. *Plant Physiol.* 173 1594–1605. 10.1104/pp.16.01907 28108698 PMC5338679

[B43] Mendes A. Kelly A. A. van Erp H. Shaw E. Powers S. J. Kurup S. (2013). bZIP67 regulates the omega-3 fatty acid content of *Arabidopsis* seed oil by activating fatty acid desaturase3. *Plant Cell* 25 3104–3116. 10.1105/tpc.113.116343 23995083 PMC3784602

[B44] Movahedi S. Van Bel M. Heyndrickx K. S. Vandepoele K. (2012). Comparative co−expression analysis in plant biology. *Plant Cell Environ.* 35 1787–1798. 10.1111/j.1365-3040.2012.02517.x 22489681

[B45] Nemhauser J. L. Hong F. Chory J. (2006). Different plant hormones regulate similar processes through largely nonoverlapping transcriptional responses. *Cell* 126 467–475. 10.1016/j.cell.2006.05.050 16901781

[B46] Ogata H. Goto S. Sato K. Fujibuchi W. Bono H. Kanehisa M. (1999). KEGG: Kyoto encyclopedia of genes and genomes. *Nucleic Acids Res.* 27 29–34. 10.1093/nar/28.1.27 9847135 PMC148090

[B47] Ohlrogge J. Browse J. (1995). Lipid biosynthesis. *Plant Cell* 7 957–970. 10.1105/tpc.7.7.957 7640528 PMC160893

[B48] Ohlrogge J. Thrower N. Mhaske V. Stymne S. Baxter M. Yang W. (2018). PlantFAdb: a resource for exploring hundreds of plant fatty acid structures synthesized by thousands of plants and their phylogenetic relationships. *Plant J.* 96 1299–1308. 10.1111/tpj.14102 30242919

[B49] O’Quin J. B. Bourassa L. Zhang D. Shockey J. M. Gidda S. K. Fosnot S. (2010). Temperature-sensitive post-translational regulation of plant omega-3 fatty-acid desaturases is mediated by the endoplasmic reticulum-associated degradation pathway. *J. Biol. Chem.* 285 21781–21796. 10.1074/jbc.M110.135236 20452984 PMC2898375

[B50] Pan X. Peng F. Y. Weselake R. J. (2015). Genome-wide analysis of *PHOSPHOLIPID: DIACYLGLYCEROL ACYLTRANSFERASE* (*PDAT*) genes in plants reveals the eudicot-wide *PDAT* gene expansion and altered selective pressures acting on the core eudicot *PDAT* paralogs. *Plant Physiol.* 167 887–904. 10.1104/pp.114.253658 25585619 PMC4348769

[B51] Pan X. Siloto R. M. P. Wickramarathna A. D. Mietkiewska E. Weselake R. J. (2013). Identification of a pair of Phospholipid:Diacylglycerol Acyltransferases from developing flax (*Linum usitatissimum* L.) seed catalyzing the selective production of trilinolenin. *J. Biol. Chem.* 288:24173. 10.1074/jbc.M113.475699 23824186 PMC3745363

[B52] Pertea G. Huang X. Liang F. Antonescu V. Sultana R. Karamycheva S. (2003). TIGR Gene Indices clustering tools (TGICL): a software system for fast clustering of large EST datasets. *Bioinformatics* 19 651–652. 10.1093/bioinformatics/btg034 12651724

[B53] Pfaffl M. W. Tichopad A. Prgomet C. Neuvians T. P. (2004). Determination of stable housekeeping genes, differentially regulated target genes and sample integrity: BestKeeper–Excel-based tool using pair-wise correlations. *Biotechnol. Lett.* 26 509–515. 10.1023/B:BILE.0000019559.84305.47 15127793

[B54] Qu J. Kang S. G. Hah C. Jang J.-C. (2016). Molecular and cellular characterization of GA-Stimulated Transcripts GASA4 and GASA6 in *Arabidopsis thaliana*. *Plant Sci.* 246 1–10. 10.1016/j.plantsci.2016.01.009 26993231

[B55] Rajwade A. V. Joshi R. S. Kadoo N. Y. Gupta V. S. (2016). Sequence characterization and *in silico* structure prediction of fatty acid desaturases in linseed varieties with differential fatty acid composition. *J. Sci. Food Agric.* 96 4896–4906. 10.1002/jsfa.7775 27109704

[B56] Rajwade A. V. Kadoo N. Y. Borikar S. P. Harsulkar A. M. Ghorpade P. B. Gupta V. S. (2014). Differential transcriptional activity of *SAD*, *FAD2* and *FAD3* desaturase genes in developing seeds of linseed contributes to varietal variation in α-linolenic acid content. *Phytochemistry* 98:41. 10.1016/j.phytochem.2013.12.002 24380374

[B57] Ramachandiran I. Vijayakumar A. Ramya V. Rajasekharan R. (2018). *Arabidopsis* serine/threonine/tyrosine protein kinase phosphorylates oil body proteins that regulate oil content in the seeds. *Sci. Rep.* 8:1154 10.1038/s41598-018-19311-19313 PMC5773694 29348626

[B58] Rao S. Abdelreheem M. Bhella R. Mccracken C. Hildebrand D. (2008). Characteristics of high alpha-linolenic acid accumulation in seed oils. *Lipids* 43 749–755. 10.1007/s11745-008-3207-3200 18597133

[B59] Saha S. Enugutti B. Rajakumari S. Rajasekharan R. (2006). Cytosolic triacylglycerol biosynthetic pathway in oilseeds. *Plant Physiol.* 141 1533–1543. 10.1104/pp.106.082198 16798944 PMC1533943

[B60] Shannon P. Markiel A. Ozier O. Baliga N. S. Wang J. T. Ramage D. (2003). Cytoscape: a software environment for integrated models of biomolecular interaction networks. *Genome Res.* 13 2498–2504. 10.1101/gr.1239303 14597658 PMC403769

[B61] Su Y. Li Y. Ye P. (2011). Mammalian meiosis is more conserved by sex than by species: conserved co-expression networks of meiotic prophase. *Reproduction* 142 675–687. 10.1530/REP-11-0260 21908654

[B62] Sun J. Chen M. Zhu M. Jiang Y. Meng J. Zhao D. (2018). Cloning, characterization, and expression analysis of three *FAD8* genes encoding a fatty acid desaturase from seeds of *Paeonia ostii*. *Molecules* 23:929. 10.3390/molecules23040929 29673187 PMC6017405

[B63] Tamura K. Dudley J. Nei M. Kumar S. (2007). MEGA4: molecular evolutionary genetics analysis (MEGA) software version 4.0. *Mol. Biol. Evol.* 24 1596–1599. 10.1093/molbev/msm092 17488738

[B64] Tarazona S. Garcíaalcalde F. Dopazo J. Ferrer A. Conesa A. (2011). Differential expression in RNA-seq: A matter of depth. *Genome Res.* 21:2213. 10.1101/gr.124321.111 21903743 PMC3227109

[B65] Thambugala D. Duguid S. Loewen E. Rowland G. Booker H. You F. M. (2013). Genetic variation of six desaturase genes in flax and their impact on fatty acid composition. *Theor*. *Appl. Genet.* 126 2627–2641. 10.1007/s00122-013-2161-2162 PMC3782649 23928861

[B66] Tian B. Lu T. Xu Y. Wang R. Chen G. (2019). Identification of genes associated with ricinoleic acid accumulation in *Hiptage benghalensis* via transcriptome analysis. *Biotechnol. Biofuels* 12:16 10.1186/s13068-019-1358-1352 PMC6340187 30679955

[B67] Troncoso-Ponce M. A. Kilaru A. Cao X. Durrett T. P. Fan J. Jensen J. K. (2011). Comparative deep transcriptional profiling of four developing oilseeds. *Plant J.* 68:1014. 10.1111/j.1365-313X.2011.04751.x 21851431 PMC3507003

[B68] Turchetto-Zolet A. C. Christoff A. P. Kulcheski F. R. Loss-Morais G. Margis R. Margis-Pinheiro M. (2016). Diversity and evolution of plant diacylglycerol acyltransferase (DGATs) unveiled by phylogenetic, gene structure and expression analyses. *Genet. Mol. Biol.* 39 524–538. 10.1590/1678-4685-gmb-2016-2024 27706370 PMC5127155

[B69] Vandesompele J. De Preter K. Pattyn F. Poppe B. Van Roy N. De Paepe A. (2002). Accurate normalization of real-time quantitative RT-PCR data by geometric averaging of multiple internal control genes. *Genome Biol.* 3 1–12. 10.1186/gb-2002-3-7-research0034 12184808 PMC126239

[B70] Voelker T. Kinney A. J. (2001). Variations in the biosynthesis of seed-storage lipids. *Annu. Rev. Plant Biol.* 52 335–361. 10.1146/annurev.arplant.52.1.335 11337402

[B71] Wang X. Liu A. (2014). Expression of genes controlling unsaturated fatty acids biosynthesis and oil deposition in developing seeds of Sacha Inchi (*Plukenetia volubilis* L.). *Lipids* 49 1019–1031. 10.1007/s11745-014-3938-z 25119487

[B72] Wong Y. C. Teh H. F. Mebus K. Ooi T. E. K. Kwong Q. B. Koo K. L. (2017). Differential gene expression at different stages of mesocarp development in high- and low-yielding oil palm. *BMC Genomics* 18:470 10.1186/s12864-017-3855-3857 PMC5480177 28637447

[B73] Xiu Y. Wu G. Tang W. Peng Z. Bu X. Chao L. (2018). Oil biosynthesis and transcriptome profiles in developing endosperm and oil characteristic analyses in *Paeonia ostii* var. *lishizhenii*. *J. Plant Physiol.* 228 121–133. 10.1016/j.jplph.2018.05.011 29902680

[B74] Xu Y. Holic R. Li D. Pan X. Mietkiewska X. Chen G. (2018). Substrate preferences of long-chain acyl-CoA synthetase and diacylglycerol acyltransferase contribute to enrichment of flax seed oil with α-linolenic acid. *Biochem. J.* 475 1473–1489. 10.1042/BCJ20170910 29523747

[B75] Yeap W. Lee F. Shabari Shan D. K. Musa H. Appleton D. R. Kulaveerasingam H. (2017). WRI 1−1, ABI 5, NF−YA 3 and NF−YC 2 increase oil biosynthesis in coordination with hormonal signaling during fruit development in oil palm. *Plant J.* 91 97–113. 10.1111/tpj.13549 28370622

[B76] Yin D. Xu W. Shu Q. Li S. Wu Q. Feng C. (2018). Fatty acid desaturase 3 (*PsFAD3*) from *Paeonia suffruticosa* reveals high α-linolenic acid accumulation. *Plant Sci.* 274 212–222. 10.1016/j.plantsci.2018.05.027 30080606

[B77] Yu S. Du S. Yuan J. Hu Y. (2016). Fatty acid profile in the seeds and seed tissues of *Paeonia* L. species as new oil plant resources. *Sci. Rep.* 6:26944. 10.1038/srep26944 27240678 PMC4886256

[B78] Yurchenko O. Park S. Ilut D. C. Inmon J. J. Millhollon J. C. Liechty Z. S. (2014). Genome-wide analysis of the omega-3 fatty acid desaturase gene family in *Gossypium*. *BMC Plant Biol.* 14 312–312. 10.1186/s12870-014-0312-315 25403726 PMC4245742

[B79] Zang X. Geng X. Ma L. Wang N. Pei W. Wu M. (2019). A genome-wide analysis of the phospholipid: diacylglycerol acyltransferase gene family in *Gossypium*. *BMC Genomics* 20:402 10.1186/s12864-019-5728-5728 PMC6530137 31117950

[B80] Zhan J. Thakare D. Ma C. Lloyd A. Nixon N. M. Arakaki A. M. (2015). RNA sequencing of laser-capture microdissected compartments of the maize kernel identifies regulatory modules associated with endosperm cell differentiation. *Plant Cell* 27 513–531. 10.1105/tpc.114.135657 25783031 PMC4558669

[B81] Zhang J. Zhang S. Zhang Y. Kitajima K. (2015). Effects of phylogeny and climate on seed oil fatty acid composition across 747 plant species in China. *Ind. Crops Prod.* 63 1–8. 10.1016/j.indcrop.2014.10.045

[B82] Zhang Q. Yu R. Sun D. Rahman M. Xie L. Hu J. (2019). Comparative transcriptome analysis reveals an efficient mechanism of α-linolenic acid in tree peony seeds. *Int. J. Mol. Sci.* 20:65. 10.3390/ijms20010065 30586917 PMC6337502

[B83] Zhang Q. Yu R. Xie L. Rahman M. M. Kilaru A. Niu L. (2018). Fatty acid and associated gene expression analyses of three tree peony species reveal key genes for α-linolenic acid synthesis in seeds. *Front. Plant Sci.* 9:106. 10.3389/fpls.2018.00106 29459881 PMC5807371

[B84] Zhang W. Chen R. Yang T. Xu N. Chen J. Gao Y. (2018). Fatty acid transporting proteins: Roles in brain development, aging, and stroke. *Prostaglandins Leukotrienes Essent. Fatty Acids* 136 35–45. 10.1016/j.plefa.2017.04.004 28457600 PMC5650946

[B85] Zhao X. Jiang H. Feng L. Qu Y. Teng W. Qiu L. (2019). Genome-wide association and transcriptional studies reveal novel genes for unsaturated fatty acid synthesis in a panel of soybean accessions. *BMC Genomics* 20:68. 10.1186/s12864-019-5449-z 30665360 PMC6341525

[B86] Zhou J. Chi X. Cheng M. Huang X. Liu X. Fan J. (2019). Zika virus degrades the ω-3 fatty acid transporter Mfsd2a in brain microvascular endothelial cells and impairs lipid homeostasis. *Sci. Adv.* 5:eaax7142. 10.1126/sciadv.aax7142 31681849 PMC6810275

[B87] Zhou S. Chen Y. Guo C. Qi J. (2020). PhyloMCL: Accurate clustering of hierarchical orthogroups guided by phylogenetic relationship and inference of polyploidy events. *Methods Ecol. Evol.* 11 943–954. 10.1111/2041-210X.13401

