# Supplementary material for: Interspecific Variation in the Unsaturation Level of Seed Oils Were Associated With the Expression Pattern Shifts of Duplicated Desaturase Genes and the Potential Role of Other Regulatory Genes
Source: Front Plant Sci. 2021 Jan 13;11:616338. doi: 10.3389/fpls.2020.616338 (PMC7838364; doi:10.3389/fpls.2020.616338)
Supplement: Supplementary file 2 [file Data_Sheet_1.docx]

Supplementary Material

## Supplementary Figures


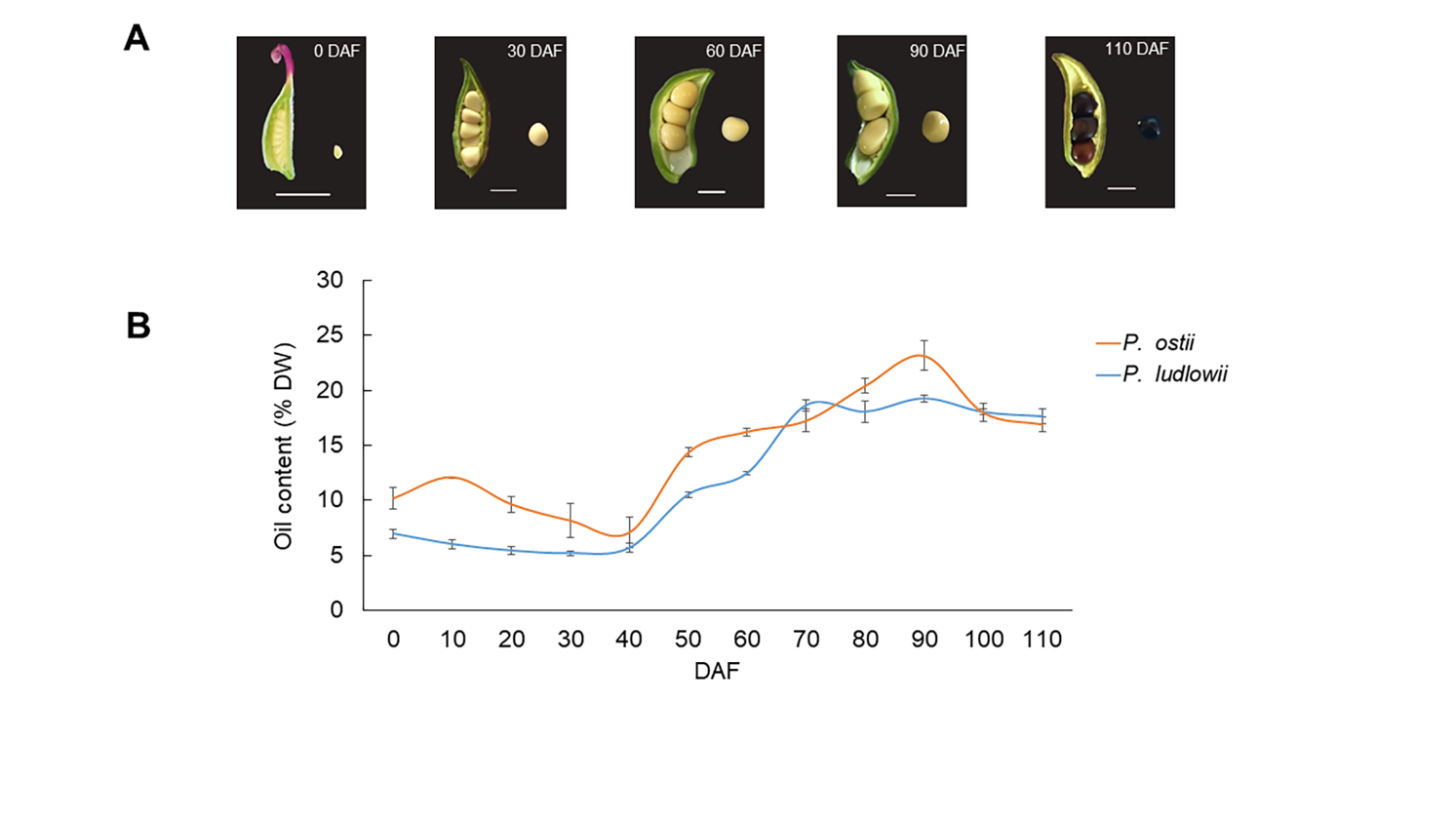


**Supplementary Figure S1.** Changes in morphology and oil content in developing tree peony seeds. **(A)** Morphological characteristics of tree peony seeds at different developmental stages. Scale bar:1 cm; **(B)** Seed-oil contents of *P. ostii* and *P. ludlowii* at different developmental stages. Quoted values are means ± SE (*N* = 3). DAF: days after flowering.


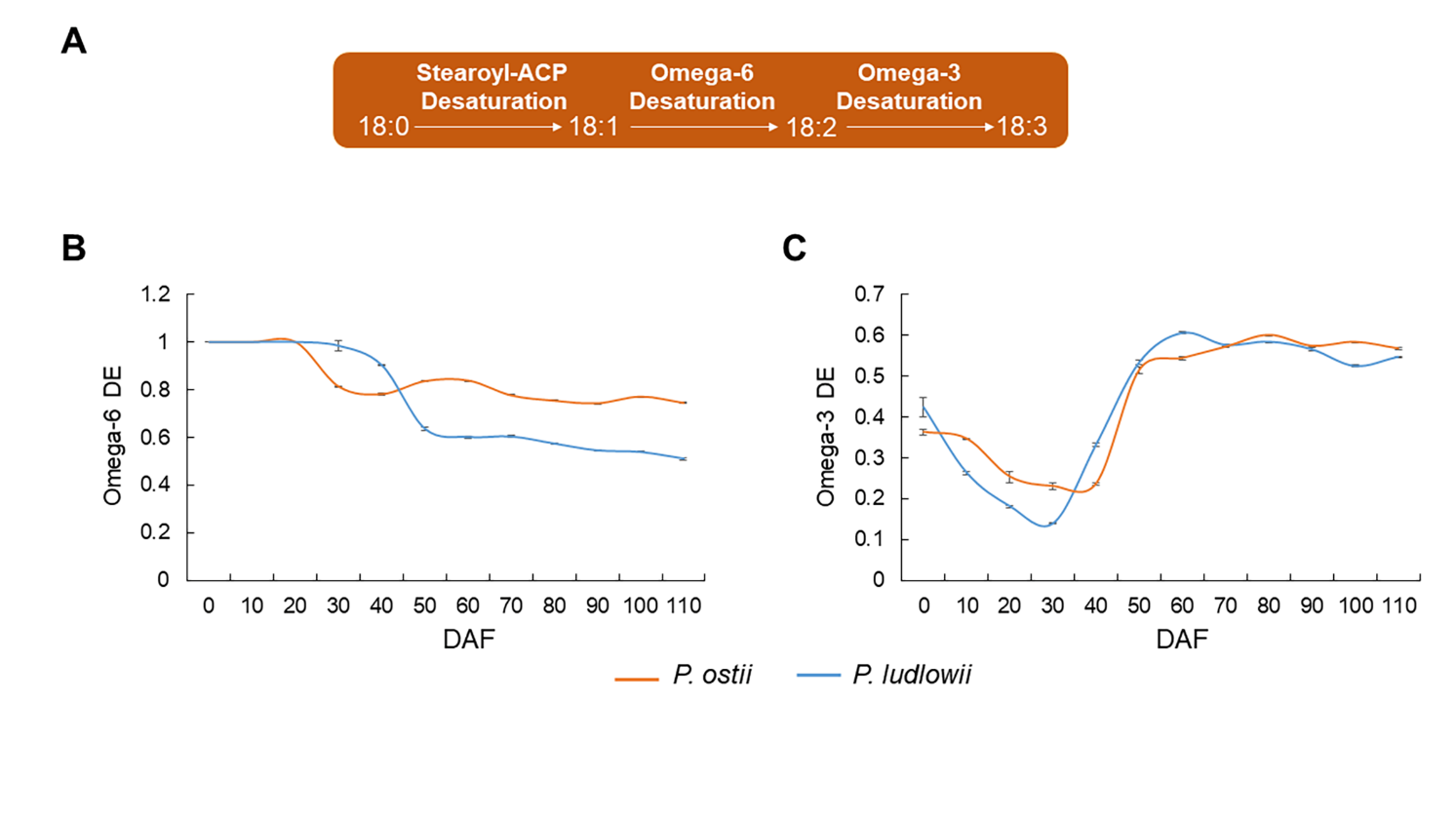


**Supplementary Figure S2.** Desaturation efficiency of desaturation reactions. **(A)** Polyunsaturated fatty acid synthesis steps; **(B)** omega-6 desaturation efficiency (DE), calculated as omega-6 DE= (18:2+18:3)/(18:1+18:2+18:3); **(C)** omega-3 desaturation efficiency(DE), calculated as omega-3 DE=18:3/(18:2+18:3). Quoted values are means ± SE (*N* = 3).


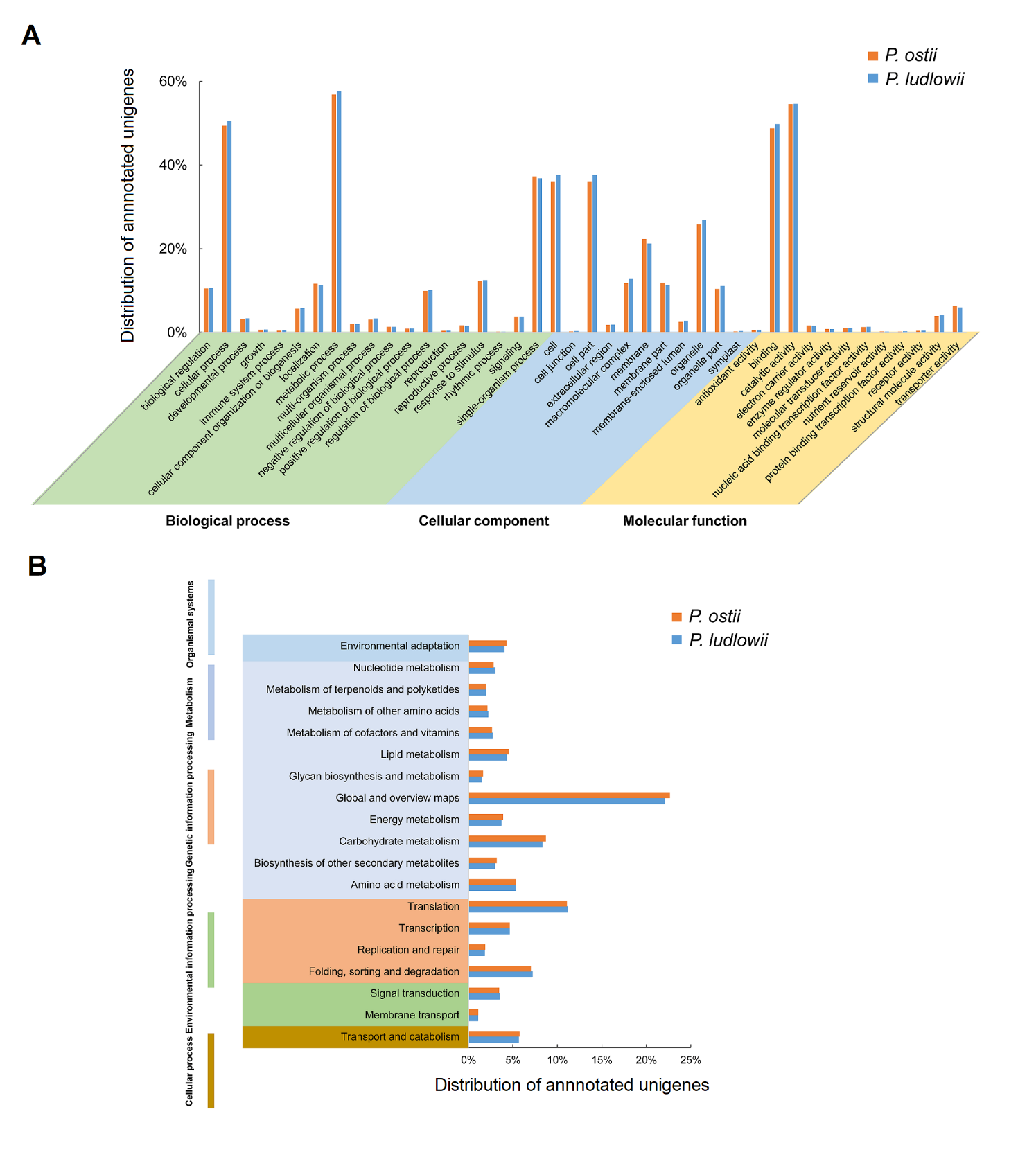


**Supplementary Figure S3.** The relative distribution of annotated unigenes. **(A)** Distribution of unigenes in Gene Ontology (GO) functional groups; **(B)** Distribution of unigenes in KEGG functional groups.


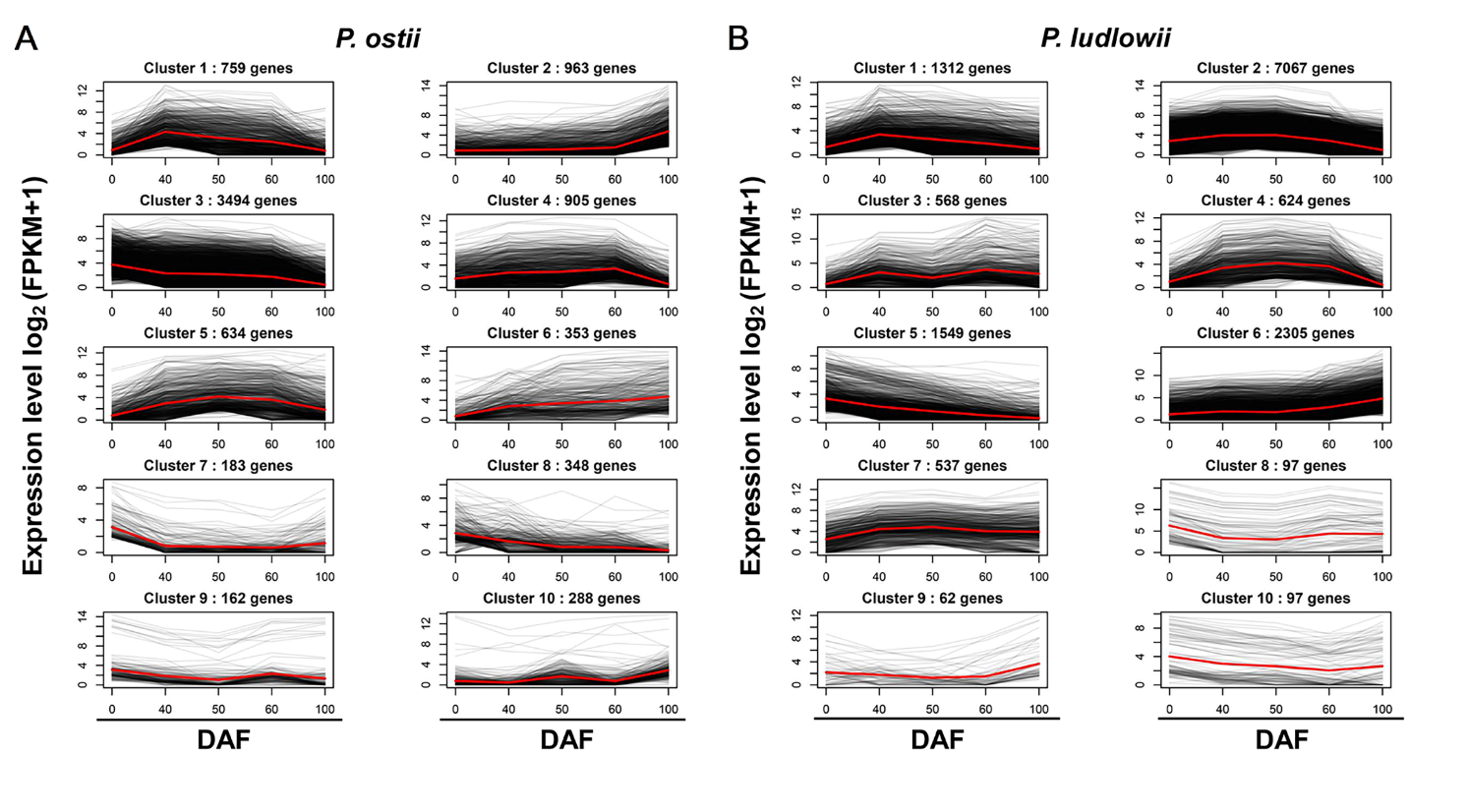


**Supplementary Figure S4.** Hierarchical clustering analysis of DEGs based on log ratio FPKM data. **(A)** Line plot for 10 clusters in *P.ostii*; **(B)** Line plot for 10 clusters in *P. ludlowii*.


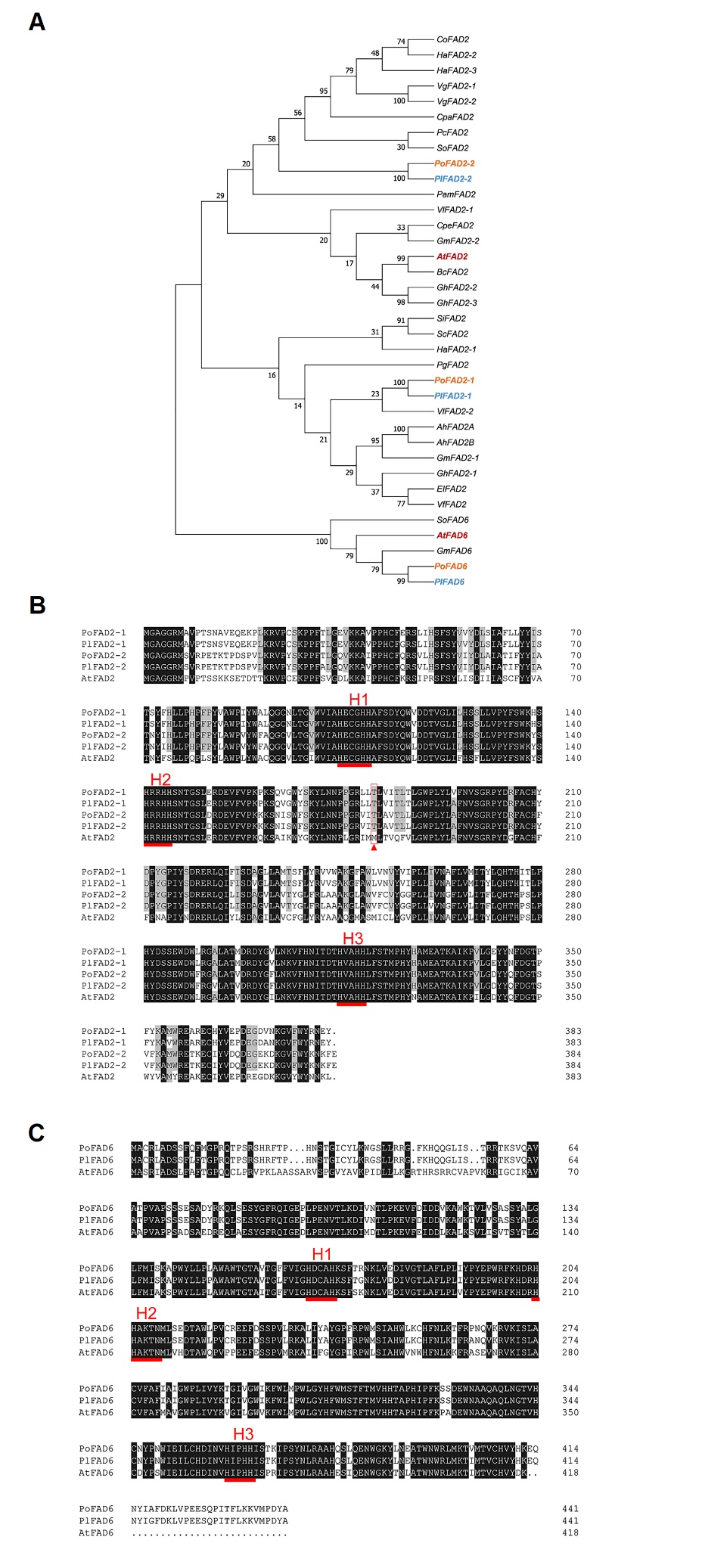


**Supplementary Figure S5.** Sequence analysis of plant omega-6 desaturase genes. (**A**) Phylogenetic analysis of FAD2 and FAD6 desaturases. Accession numbers of the sequences included are: *Calendula officinalis* (*CoFAD2*, AF343065), *Helianthus annuus* (*HaFAD2-1*, AF251842; *HaFAD2-2*, AF251843; *HaFAD2-3*, AF251844), *Vernonia galamensis* (*VgFAD2-1*, AF188263; *VgFAD2-2*, AF188264), *Crepis palestina* (*CpaFAD2*, Y16284), *Petroselinum crispum* (*PcFAD2*, U86072), *Spinacia oleracea* (*SoFAD2*, AB094415; *SoFAD6*, X78311), *Persea americana* (*PamFAD2*, AY057406), *Vitis labrusca* (*VlFAD2-1*, JF429694; *VlFAD2-2*, JF429695), *Cucurbita pepo* (*CpeFAD2*, AY525163), *Arabidopsis thaliana* (*AtFAD2*, L26296; *AtFAD6*, U09503), *Brassica carinata* (*BcFAD2*, AF124360), *Arachis hypogaea* (*AhFAD2A*, AF030319; *AhFAD2B*, AF272950), *Glycine max* (*GmFAD2-1*, L43920; *GmFAD2-2*, L43921; *GmFAD6*, L29215), *Gossypium hirsutum* (*GhFAD2-1*, X97016; *GhFAD2-2*, Y10112; *GhFAD2-3*, AF331163), *Sesamum indicum* (*SiFAD2*, AF192486), *Euphorbia lagascae* (*ElFAD2*, AY486148), *Vernicia fordii* (*VfFAD2*, AF525535), *Solanum commersonii* (*ScFAD2*, X92847), and *Punica granatum* (*PgFAD2*, AJ437139); **(B)** Alignment of the FAD2 amino acid sequences of different species, showing the conserved histidine boxes (underlined with red lines) and the residue possibly susceptible to phosphorylation (marked with a red triangle); **(C)** Alignment of the FAD6 amino acid sequences of different species, showing the conserved histidine boxes (underlined with red lines).


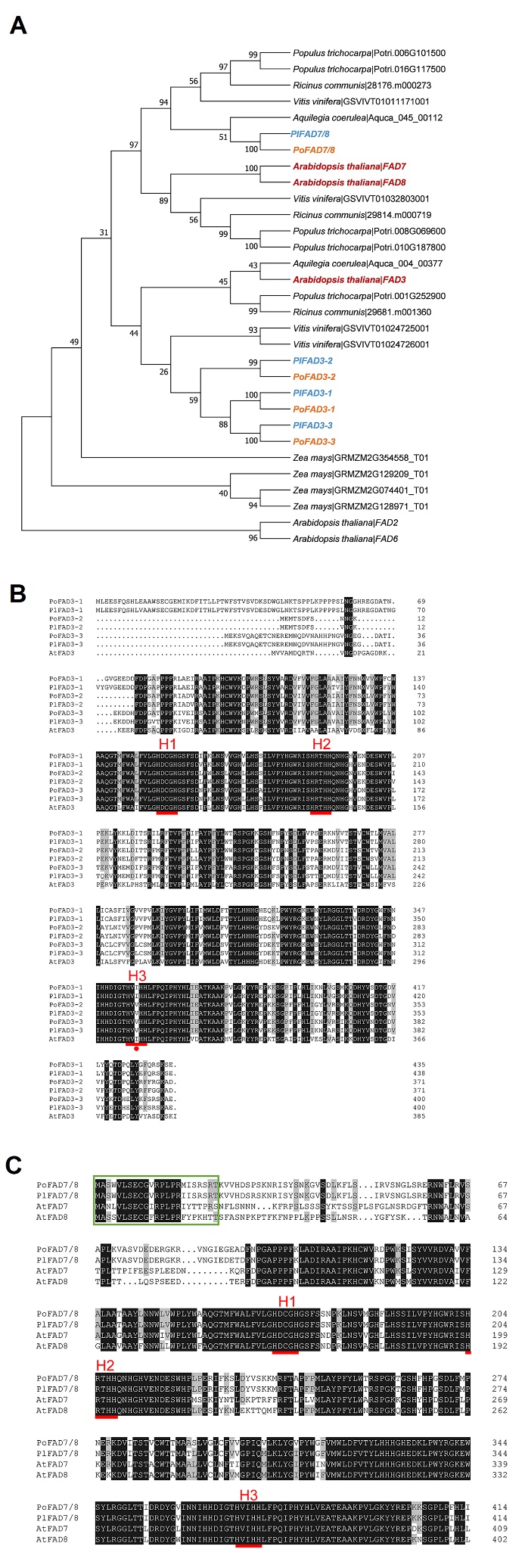


**Supplementary Figure S6.** Sequence analysis of plant omega-3 desaturase genes. **(A)** Phylogenetic analysis of the FAD3 and FAD7/8 desaturases of different species, including *Aquilegia coerulea*, *Arabidopsis thaliana*, *Populus trichocarpa*, *Ricinus communis*, *Vitis vinifera*, *Zea mays*; **(B)** Alignment of the FAD3 amino acid sequences of different species, showing three characteristic histidine boxes (underlined with red lines) and the amino acid residue substitution occurred within the conserved histidine box (marked with a red dot); **(C)** Alignment of the FAD7/8 amino acid sequences of different species, showing the conserved histidine boxes (underlined with red lines) and the plastid-targeting peptides (marked with a square of green lines).


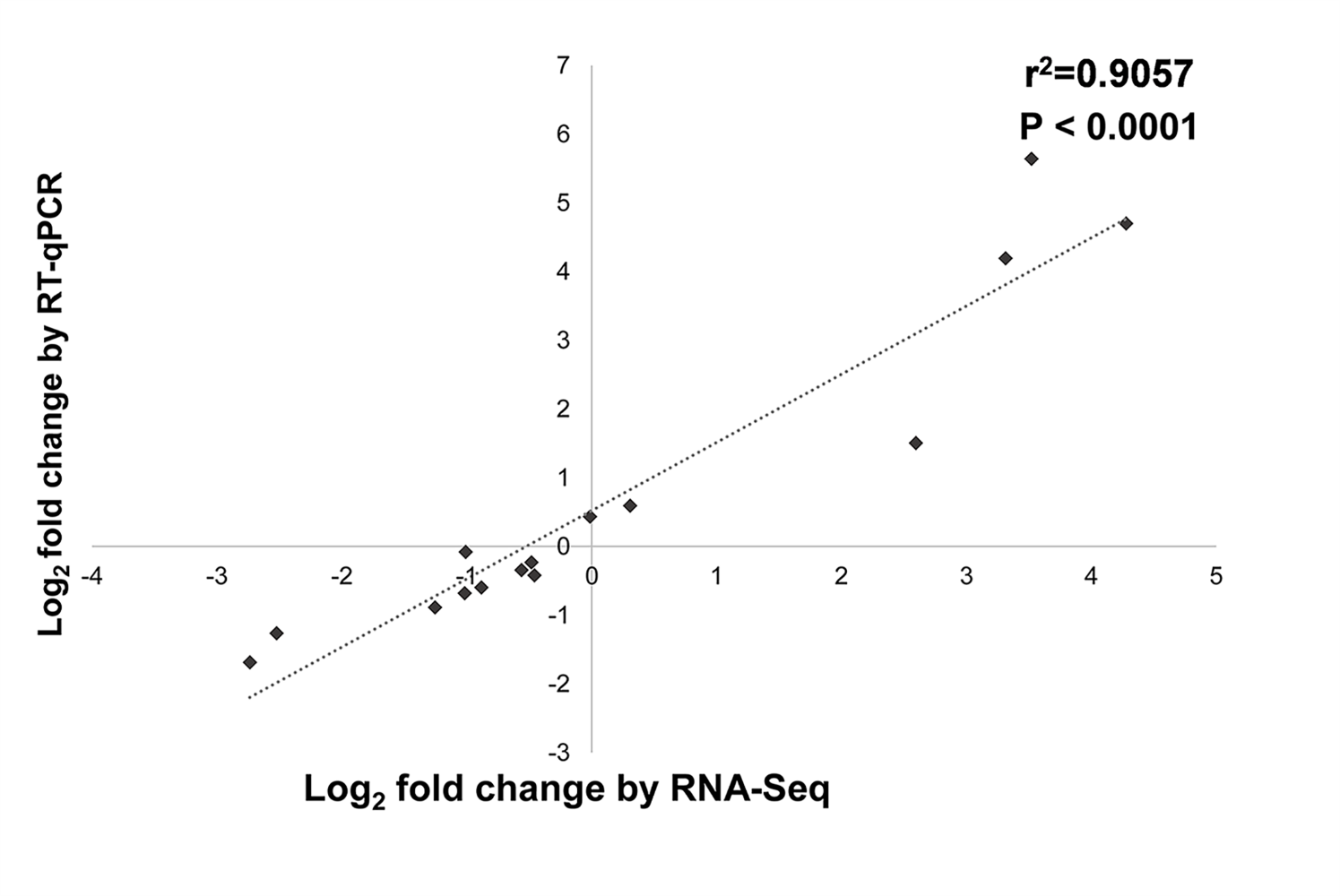


**Supplementary Figure S7.** Scatter plot showing correlation between RNA-Seq RT-qPCR expression profiles. P value and r^2^ value of Pearson correlation were calculated between the log2 fold change values of expression levels of 15 genes from *P. ostii* and *P. ludlowii* inferred by RNA-Seq and RT-qPCR, respectively (at 50 DAF).


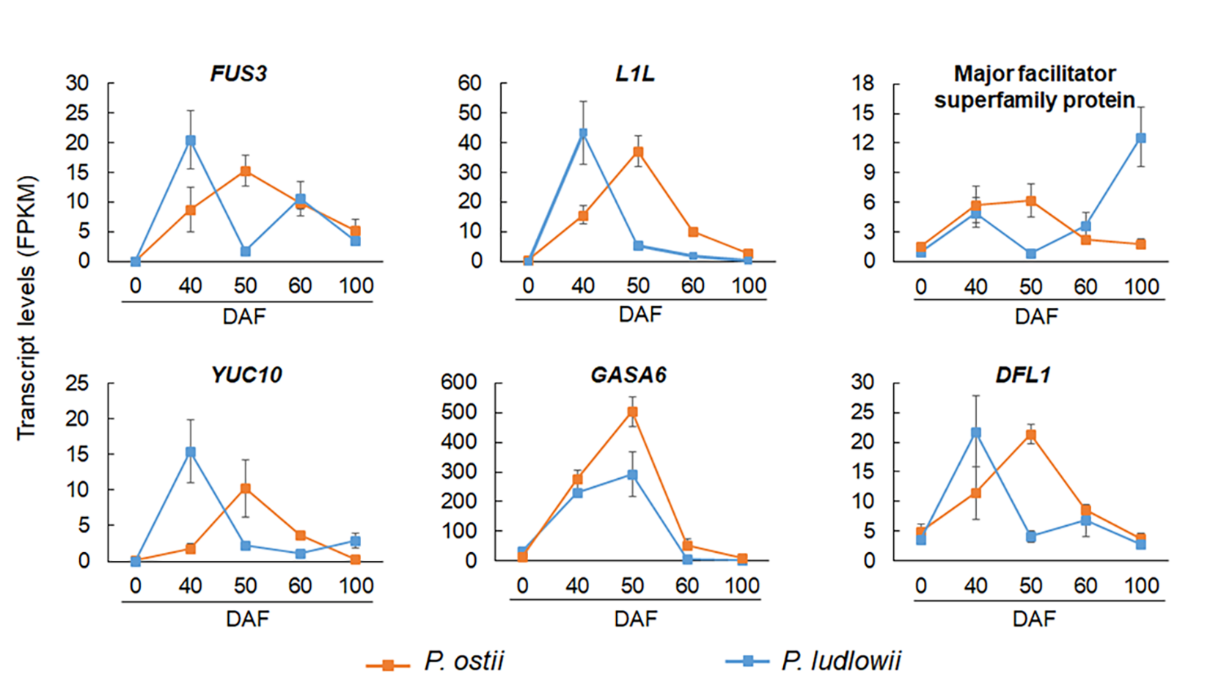


**Supplementary Figure S8.** The candidate genes associated with *FAD2* and *FAD3* were differentially expressed between *P. ostii* and *P. ludlowii*. Error bars indicate the SE of the mean.
